# Circadian actions of orexins on the retinorecipient lateral geniculate complex in rat

**DOI:** 10.1113/JP280275

**Published:** 2020-10-14

**Authors:** Lukasz Chrobok, Jagoda Stanislawa Jeczmien‐Lazur, Kamil Pradel, Jasmin Daniela Klich, Monika Bubka, Michal Wojcik, Mariusz Kepczynski, Marian Henryk Lewandowski

**Affiliations:** ^1^ Department of Neurophysiology and Chronobiology Institute of Zoology and Biomedical Research Jagiellonian University in Krakow Krakow Poland; ^2^ Department of Glycoconjugate Biochemistry Institute of Zoology and Biomedical Research Jagiellonian University in Krakow Krakow Poland; ^3^ Department of Physical Chemistry and Electrochemistry Faculty of Chemistry Jagiellonian University in Krakow Krakow Poland

**Keywords:** circadian clock, lateral geniculate nucleus, multi‐channel electrophysiology, neuronal tract tracing, optogenetics, orexins, PAC1 receptor, retinorecipient, subcortical visual system

## Abstract

**Key points:**

- Rhythmic processes in living organisms are controlled by biological clocks. The orexinergic system of the lateral hypothalamus carries circadian information to provide arousal for the brain during the active phase.
- Here, we show that orexins exert an excitatory action in three parts of the lateral geniculate nucleus (LGN), in particular upon directly retinorecipient neurons in the non‐image forming visual structures.
- We provide evidence for the high nocturnal levels of orexins with stable circadian expression of predominant orexin receptor 2 in the LGN.
- Our data additionally establish the convergence of orexinergic and pituitary adenylate cyclase (PAC)‐activating peptide/PAC1 receptor systems (used by melanopsin‐expressing retinal ganglion cells), which directly regulates responses to the retinal input.
- These results help us better understand circadian orexinergic control over the non‐image forming subcortical visual system, forming the animal's preparedness for the behaviourally active night.

**Abstract:**

The orexinergic system of the lateral hypothalamus is tightly interlinked with the master circadian clock and displays daily variation in activity to provide arousal‐related excitation for the plethora of brain structures in a circadian manner. Here, using a combination of electrophysiological, optogenetic, histological, molecular and neuronal tracing methods, we explore a particular link between orexinergic and visual systems in rat. The results of the present study demonstrate that orexinergic fibre density at the area of subcortical visual system exerts a clear day to night variability, reaching a maximum at behaviourally active night. We also show pronounced electrophysiological activations of neurons in the lateral geniculate nucleus by orexin A through 24 h, via identified distinct orexin receptors, with the ventrolateral geniculate displaying a daily cycle of responsiveness. In addition, for the first time, we provide a direct evidence for orexins to act on retinorecipient neurons with a high convergence of orexinergic and putatively retinal pituitary adenylate cyclase (PAC)‐activating peptide/PAC1 receptor systems. Altogether, the present study ties orexins to non‐image forming visual structures with implications for circadian orexinergic modulation of neurons, which process information on ambient light levels.

## Key points

- Rhythmic processes in living organisms are controlled by biological clocks. The orexinergic system of the lateral hypothalamus carries circadian information to provide arousal for the brain during the active phase.
- Here, we show that orexins exert an excitatory action in three parts of the lateral geniculate nucleus (LGN), in particular upon directly retinorecipient neurons in the non‐image forming visual structures.
- We provide evidence for the high nocturnal levels of orexins with stable circadian expression of predominant orexin receptor 2 in the LGN.
- Our data additionally establish the convergence of orexinergic and pituitary adenylate cyclase (PAC)‐activating peptide/PAC1 receptor systems (used by melanopsin‐expressing retinal ganglion cells), which directly regulates responses to the retinal input.
- These results help us better understand circadian orexinergic control over the non‐image forming subcortical visual system, forming the animal's preparedness for the behaviourally active night.

## Introduction

Most, if not all living organisms organize their physiological processes according to rhythmic environmental cues with a period of circa 24 h, as a consequence of specialized molecular mechanisms termed biological clocks (Hastings *et al*. 2018; Challet, 2019). In mammals, the central circadian pacemaker is localized in the suprachiasmatic nucleus (SCN) of the hypothalamus, which is sensitive to ambient light levels as a result of rich innervation from the intrinsically photosensitive retinal ganglion cells (ipRGCs) expressing a ‘circadian photopigment’ melanopsin (Hattar *et al*. 2002; Schmidt *et al*. 2011). Similar to the SCN not being the only autonomous brain clock, it also does not represent the only brain site receiving light information from ipRGCs (Guilding & Piggins, 2007; Albrecht, 2012). The subcortical visual system additionally includes other retinorecipient brain structures in the thalamus, pretectum and tectum, with the intergeniculate leaflet (IGL), olivary pretectal nucleus (OPT) and posterior limitans nucleus (PLi) being the major recipients of ipRGC innervation alongside the SCN (Hattar *et al*. 2006). To communicate levels of environmental light, the melanopsin‐expressing cells mostly co‐release glutamate and pituitary adenylate cyclase (PAC)‐activating peptide (AP) acting through PAC1 receptors, to excite targeted neurons in the brain (Hannibal *et al*. 2000).

The orexinergic system of the lateral hypothalamus (de Lecea, 1998; Sakurai *et al*. 1998) has been shown to tightly co‐operate with the master clock, providing high levels of arousal during the active phase and being sensitive to ambient light (Peyron *et al*. 1998; McGranaghan & Piggins, 2001; Marston *et al*. 2008; Webb *et al*. 2008; Belle *et al*. 2014; Belle & Piggins, 2017; Karnani *et al*. 2020). Therefore, it has been hypothesized that orexins (orexin A and B; OXA and OXB) may act as hands of the clock for these brain systems that are not directly innervated by the SCN. Previous studies link the orexinergic system with the visual one, showing orexinergic innervation of the visual thalamus (Peyron *et al*. 1998; McGranaghan & Piggins, 2001; Mintz *et al*. 2001) and orexin sensitivity in the retina (Qiao *et al*. 2017; Zhang *et al*. 2018), three parts of the LGN (Pekala *et al*. 2011; Palus *et al*. 2015; Chrobok *et al*. 2016, 2017; Palus‐Chramiec *et al*. 2019; Orlowska‐Feuer *et al*. 2019) and the visual cortex (Bayer *et al*. 2004), or the SCN (Brown *et al*. 2008; Belle *et al*. 2014; Belle & Piggins, 2017). Additionally, there is some evidence for both rodent and human retina to synthesize orexins and their receptors (orexin receptor 1 and 2; OX_1_R and OX_2_R) (Savaskan *et al*. 2004; Liu *et al*. 2011).

There is a clear understanding on how the subcortical visual system receives the information on the light/dark photocycle. However, how the circadian system modulates this set of retinorecipient brain structures, especially in the thalamus, still remains elusive. Moreover, knowledge of the convergence of two peptidergic systems, comprising that of the hypothalamic orexinergic and the retinal PACAP/PAC1 receptor systems on retinorecipient neurons in the thalamus, has vast implications on the role of circadian timekeeping with respect to the modulation of light‐directed behaviours.

Here, using a combination of *ex vivo* multi‐channel electrophysiology together with optogenetic, molecular, immunohistochemical and tract tracing methods, we tackle the issue of orexin sensitivity of the LGN complex throughout the day and night. Because previous experiments were mostly performed on young animals (Bayer *et al*. 2002; Govindaiah & Cox, 2006; Palus *et al*. 2015; Chrobok *et al*. 2016, 2017; Palus‐Chramiec *et al*. 2019), the present study only focuses on adult rats. This is particularly important because studies on pups have resulted in major interpretation discrepancies, such as attributing the role of orexin signalling in the dorso‐lateral geniculate (DLG) to vision modulation, rather than to the development of visual circuits. The results of the present study show that the orexinergic system of the hypothalamus exerts a powerful excitatory influence upon the non‐image forming parts of the adult LGN, including directly retinorecipient neurons, with a higher ligand presence during the night and stable expression of predominant OX_2_R throughout 24 h. Moreover, we characterize the convergence of orexinergic and PACAP/PAC1 receptor systems, and show the activation of PAC1 receptor with respect to modulating the responsiveness to retinal input. Altogether, the present study provides a comprehensive description of the daily action of orexinergic system to excite retinorecipient neurons in the non‐image forming visual structures, with implications for their modulation by arousal in the circadian fashion. All of these factors maximize an animal's preparedness for the behaviourally active night.

## Methods

### Ethical approval

All procedures were approved by the Local Ethics Committee of the Jagiellonian University in Krakow. Animals were maintained and used in accordance with Polish regulations and the European Communities Council Directive (86/609/EEC). All procedures were designed to minimize the number of animals and their sufferings. This work complies with the principles and regulations as described in the editorial by Grundy (2015).

### Animals

All experiments were performed on 85 adult male Sprague–Dawley rats kept under a standard light/dark photocycle (12:12 h LD) with access to water and food available *ad lib*., bred in house by the Institute of Zoology and Biomedical Research Animal Facility at the Jagiellonian University in Krakow. Animals were housed two to six per cage at 23 ± 2°C and 67 ± 5% relative humidity.

### Intraocular injections

#### Surgery

Twelve adult Sprague–Dawley rats (10–12 weeks old) were deeply anaesthetized with isoflurane (3% v/v air mixture; Baxter, Deerfield, IL, USA) in a closed chamber (Isoflurane Vaporizer; Stoelting, Wood Dale, IL, USA). Next, animals were placed in a mask which allowed them to breathe the air containing 3% isoflurane throughout the procedure. Rats were then subjected to i.m. injections of Torbugesic (0.2 mg kg^−1^ body weight; Zoetis, Parsippany, NJ, USA) and Tolfedine 4% (4 mg kg^−1^ body weight; Biowet, Puławy, Poland). Intraocular injections of 0.5% cholera toxin b (CtB) (2 µL; Sigma, Darmstadt, Germany; *n* = 7 rats) or AAV2‐Syn‐Chronos‐green fluorescent protein (GFP) (2–3 µl, 2.1 × 10^12^ virus molecules mL^−1^; UNC Vector Core, Chapel Hill, NC, USA; *n* = 5 rats) diluted in saline were performed with a 5 µL Hamilton syringe through a 30 G injection needle into the vitreous chamber of one eye. Animals were returned to the animal facility and monitored every day for signs of distress. To avoid infection, Sul‐Tridin 24% (sulphadiazine 200 mg mL^−1^ + trimethoprim 40 mg mL^−1^; ScanVet, ScanVet, Poland, Poland) dissolved in drinking water (1:300) was implemented for the first week post‐surgery. These animals which were injected with the adeno‐associated virus (AAV) were used in electrophysiological procedures after 4–5 weeks, whereas these injected with CtB were subjected to immunohistological studies after 3 days.

#### Immunostaining and imaging

CtB injected rats were re‐anaesthetized with isoflurane (2 mL kg^−1^ body weight) and i.p. injected with sodium pentobarbital (100 mg kg^−1^ body weight; Biowet). Then, rats were submitted to a tissue fixation process by transcardial perfusion with 4% paraformaldehyde (PFA) in 0.1 m PBS. Next, brains were removed from the skull and post‐fixed in the same fixative solution overnight. Tissue was cut on a vibroslicer (VT1000S; Leica Microsystems, Wetzlar, Germany) in 40 µm thick coronal thalamic/pretectal sections. After sectioning, slices were transferred to a permeabilizing solution containing 0.5% Triton‐X100 (Sigma) and 5% normal donkey serum (NDS) (Jackson ImmunoResearch, West Grove, PA, USA) in PBS at room temperature. After 35 min, slices were washed off in PBS and incubated with primary antisera against OXB (raised in goat, dilution 1:500; Santa Cruiz Biotechnology, Santa Cruiz, CA, USA) and CtB (raised in mouse, dilution 1:250; Abcam, Cambridge, UK) in PBS containing 0.5% NDS and 0.1% Triton‐X100 overnight at 4°C. In the subsequent step, slices were washed off twice in fresh PBS and incubated overnight at 4°C with fluorochrome‐conjugated secondary antisera (dilution 1:300): donkey anti‐goat Alexa Fluor 647 and donkey anti‐mouse Cy3. Finally, sections were mounted on glass slides, dried and coverslipped with Fluoroshield (Sigma). Confocal images were collected using an A1Si (Nikon, Tokyo, Japan) confocal laser scanning system built on an inverted Ti‐E microscope (Nikon).

### Immunohistochemical staining across 24 h

#### Tissue collection and immunohistochemical staining

Twenty‐four adult (12–16 weeks old) male Sprague–Dawley rats were anaesthetized and transcardially perfused with the 4% PFA (see ‘Intraocular injections – Immunostaining and imaging’ above) throughout the 24 h at four time points: five at zeitgeber time (ZT)0, five at ZT6, six at ZT12 and six at ZT18. Brains were excised, post‐fixed and cut on the vibroslicer, accordingly. Sections containing the OPT and PLi were immunostained with anti‐parvalbumin (PVA) (dilution 1:1000; Abcam) and anti‐OXB (dilution 1:500) primary antisera. Brain slices comprising the LGN were immunostained for neuropeptide Y (NPY), instead of PVA, with primary antibodies raised in rabbit (dilution 1:8000; Sigma) and for OXB. Next, all slices were incubated with Cy3‐conjugated secondary donkey anti‐rabbit and Alexa Fluor 647‐conjugated donkey anti‐goat antisera (both dilution 1:300; Jackson ImmunoResearch). Slices were then mounted and imaged under a confocal microscope.

#### Imaging and analysis

The area containing the OPT, PLi and LGN was scanned under a 20× objective in 3 µm Z‐stack steps. Areas were identified in accordance with the marker staining for PVA or NPY. Images were further analysed using the Fiji Software (NIH, Bethesda, MD, USA) for optimal data representation, with the use of custom made macro. First, the Bernsen's adaptive thresholding method was used to define regions of high local contrast. Next, immunoreactive (ir) pixels representing OXB‐ir fibres were automatically counted in the regions of interest (ROIs) outlined by a 40 × 40 pixel oval, and then divided by the area of the measurement to represent the area fraction. Three ROIs were measured in each image and the area fraction values were averaged. For the OPT and PLi, two slices were analysed from each animal and values were again averaged. For the LGN, four slices were measured for each rat (two caudal and two rostral) and all were averaged. All of the digital images were processed with the same settings.

### Neuronal tract tracing

#### Surgery

Five adult male Sprague–Dawley rats (250–350 g) were anaesthetized and prepared for surgery as described above for ‘Intraocular injections – Surgery’. During the surgery, rats were mounted in the Small Animal Stereotaxic System (SAS‐4100; ASI Instruments, Warren, MI, USA) equipped with a rat gas anaesthesia mask (Stoelting). The core body temperature was maintained at 37°C using an automatic small animal heating pad (WMT, Krakow, Poland). Retrograde tracers (FluoroGreen and FluoroRed; 20 nL; Tombow Pencil Co., Tokyo, Japan) were injected into the LGN (centred at IGL: anteroposterior = −4.7, mediolateral = ±3.8, dorsoventral = −4.9 mm from Bregma) with the 1 µL Hamilton syringe connected to the microinjection needle pulled from borosilicate glass capillaries (Sutter Instruments, Novato, CA, USA). To ensure proper wound healing, stitched skin was secured with anti‐bacterial ointment (Tribiotic; Kato, Wasaw, Poland) and the drinking water was supplemented with Sul‐Tridin 24% (dilution 1:300). Rats were monitored every day for signs of post‐surgery distress. After 7 days of recovery, rats were re‐anaesthetized and the same procedure was used to perform an unilateral injection of colchicine (0.1 mg in 5 µL of saline; Sigma) into the lateral ventricle (anteroposterior = −0.7, mediolateral = +1.8, dorsoventral = −4 mm from Bregma) to improve the somatic OXB immunostaining. After 24 h, rats were deeply anaesthetized and perfused.

#### Tissue collection, immunostaining and imaging

After 24 h post‐fixation process in the 4% PFA, brains were cut in the chamber of a vibroslicer into 40 µm slices containing the lateral hypothalamic area (every third slice was collected) or 100 µm thalamic sections (for tracer injection site visualization). Hypothalamic slices were immunostained for OXB only, following the same protocol as described above for ‘Intraocular injections – Immunostaining and imaging’ and then examined with the epifluorescence microscope (Axio Imager M2; Carl Zeiss, Oberkochen, Germany).

### Electrophysiological recording *ex vivo*

#### Tissue preparation

In total, 25 adult Sprague–Dawley rats (10–12 weeks old) were used in the present study, with five culled at ZT0, five at ZT6, five at ZT12 and five at ZT18 for recordings examining day to night variation in neuronal activity and responsiveness to OXA, as well as five at ZT1 for optogenetic studies (10 weeks old). The brain was quickly removed from the skull and immersed in the ice cold preparation artificial cerebro‐spinal fluid (ACSF), comprising (in nm): 25 NaHCO_3_, 3 KCl, 1.2. Na_2_HPO_4_, 2 CaCl_2_, 10 MgCl_2_‧6H_2_O, 10 glucose, 125 sucrose and 0.01 mg L^−1^ phenol red; oxygenated with carbogen (95% oxygen, 5% CO_2_). Next, the forebrain was carefully separated and cut on a vibroslicer into 250 µm thick slices. Slices were further processed to warm (32°C), oxygenated recording ACSF comprising (in mM): 125 NaCl, 25 NaHCO_3_, KCl 3, 1.2 Na_2_HPO_4_, 2 CaCl_2_, 2 MgCl_2_‧6H_2_O, 5 glucose and 0.01 mg L^−1^ phenol red and incubated for an hour prior to locating in the recording chamber of the multi‐electrode array (MEA) system.

#### Recording

Sections were then mounted in the recording wells of the MEA2100‐System (Multi Channel Systems, Kusterdingen, Germany). The LGN was positioned upon the recording electrodes of the 6 × 10 perforated MEA (100 µm spacing; 60pMEA100/30iR‐Ti; Multi Channel Systems). Throughout the whole recording, slices were perfused with the fresh recording ACSF (2 mL min^−1^), constantly heated to 32°C and stabilized using a gentle suction. Prior to each recording, the tissue was allowed to settle for 0.5 h. Drugs: OXA (OXA; 20, 50 or 200 nm; Bachem, Bubendorf, Switzerland), maxadilan (MAX; PAC1 receptor agonist; 50 or 100 nm; Bachem) and TCS‐OX2‐29 (TCS; OX_2_ receptor antagonist; 10 µm; Tocris, Bristol, UK) were stored as 100–1000× concentrates and were freshly diluted in the recording ACSF prior the application. All drugs were delivered by bath perfusion and around 4 min was needed for the drug to reach the recording chamber. Signal was sampled at 20 kHz and acquired with a Multi Channel Experimenter (Multi Channel Systems).

#### Optogenetic stimulation

A blue diode (PlexBright LED 465 nm controlled by a LD1 LED driver; Plexon, Dallas, TX, USA) was coupled to an optical fibre (200 µm core diameter, 0.5 NA; Thorlabs, Bergkirchen, Germany), which was placed above the recorded slice in a distance enabling the beam of blue light to cover the whole recording area. A matching light intensity (∼10 mW; measured with a photodiode power sensor S121C connected to a digital power meter PM100D; Thorlabs) and flash duration (1 ms) was used in all of the experiments. Two optogenetic stimulation protocols were applied, one after the other. First, the ‘step stimulation’ was used, consisting of 15 trains (train duration: 3 s) of an increasing flash frequency: 1, 2, 3, 5, 7, 10, 15, 20, 30, 40, 50, 60, 70, 80 and 100 Hz, divided by 5 s intervals. Subsequently, the ‘incremental stimulation’ was constructed from a train of 100 flashes appearing in an incremental (1 Hz) frequency from 1 to 100 Hz. These were repeated ten times, every 5 s. Custom made scripts written in Spike2 (Cambridge Electronic Design Ltd, Cambridge, UK) were used to apply optogenetic light stimulation protocols.

#### Spike‐sorting and single unit analysis

Raw data were exported to HDF5 files with Multi Channel DataManager (Multi Channel Systems) and subjected to a custom made MatLab script (MatLab R2018a; MathWorks Inc., Natick, MA, USA) for remapping and converting the file to a DAT format. DAT files were initially automatically spike‐sorted using KiloSort (Pachitariu *et al*. 2016) in the MatLab environment. For spike sorting speed enhancement, the GPU was used (GeForce GTX 1050Ti GPU; CUDA 9.0 for Windows; NVIDIA, Santa Clara, CA, USA). In parallel, raw data were exported to CED‐64 files with Multi Channel DataManager, then subsequently remapped and filtered with the Butterworth band pass filter (fourth order) from 0.3 to 7.5 kHz. Spike‐sorting results were transferred into the prepared CED‐64 files (Spike2 8.11; Cambridge Electronic Design Ltd) for further visualization. Manual refinement of single units was conducted using principal components analysis and autocorrelation.

For baseline analyses, single unit data were 1 s binned and explored in the 2500 s window. Periodograms and autocorrelograms were constructed and further analysis of phasic firing was performed using a custom made script in R (R Core Team 2018; R Foundation for Statistical Computing, Vienna, Austria). For the examination of drug responses, data were 30 s binned and analysed in NeuroExplorer 6 (Nex Technologies, Lenora, KS, USA). If, after the drug application, the change in single unit activity exceeded three SDs from baseline mean, a unit was classified as responsive. Response amplitudes were further calculated as the difference between the 600 s baseline mean and a maximal value during the response to a drug. For optogenetic responses, single unit data were analysed in 10 ms windows after each flash with a custom made Spike2 8.11 script measuring the spike presence (fidelity), number and latency.

### Quantitative real‐time polymerase chain reaction (qPCR)

#### Tissue preparation

In total, 24 adult (12 weeks old) male Sprague–Dawley rats were culled at four time points across 24 h (ZT0, 6, 12 and 18; *n* = 6 each). Brains followed the same slicing procedure as described above for ‘Electrophysiological recording *ex vivo* – Tissue preparation’. From the 250 µm thick thalamic sections obtained, whole bilateral LGNs were dissected using a scalpel and flash frozen upon the dry ice. Tissue fragments containing the LGN only were then stored in −80°C, for up to 3 days. During the preparation, all instruments were surface treated with RNaseZAP (Sigma) to minimize ribonuclease activity.

#### RNA extraction and real‐time qPCR

The dissected tissue was then processed for RNA extraction using the ReliaPrep RNA Tissue Miniprep System (Promega, Madison, WI, USA) and the RNA obtained was stored in water at −80°C. Reverse transcription was performed using the High‐Capacity RNA‐to‐cDNA Kit (Applied Biosystems, Foster City, CA, USA), using the same amount of RNA (200 ng) for each sample. Subsequently, real‐time PCR was performed using PowerUp SYBR Green Master Mix (ThermoFisher Scientific, Vilnius, Lithuania) and measured with the StepOnePlus Real‐Time PCR System (Applied Biosystems). Transcripts were amplified using the QuantiTect primer assay (Qiagen, Hilden, Germany) for *Hcrtr1*, *Hcrtr2* and *Adcyap* genes, with *Gapdh* as a housekeeping gene. The ΔΔCT method was used for data analysis with *Gapdh* as a reference gene and ZT0 mean values for relative target gene expression (RQ = 1).

## Statistical analysis

All statistical tests were performed in Prism, version 7 (GraphPad Software Inc., San Diego, CA, USA) and data are presented as the mean ± SD. *P* < 0.05 was considered statistically significant.

A Mann–Whitney test was used to compare the efficiency of OXA‐evoked response blockage by TCS‐OX2‐29 in the IGL *vs*. VLG.

Ordinary one‐way ANOVA was used to test: (i) the daily variability of OXB‐ir fibre density at the area of retinorecipient brain structures; (ii) differences in electrophysiological parameters of phasic/rhythmic units in three LGN subnuclei; (iii) the daily variability in gene expression; and (iv) differences in the latency of optogenetically‐evoked action potentials among three parts of the LGN. A Kruskal–Wallis test was used to assess: (i) the daily change in the electrophysiological responsiveness to OXA or MAX and (ii) variability in the firing rate of single units in three LGN subdivisions across 24 h. A Friedman test followed by Dunn's multiple comparison test was performed to verify the reduction of OXA‐evoked response by TCS‐OX2‐29 or to assess possible extinction of response to the subsequent OXA application (control treatment).

Repeated measures (RM) two‐way ANOVA was performed to verify the modulatory effect of OXA and MAX upon optogenetically‐evoked retinal responses in the LGN.

## Results

### Hypothalamic orexinergic innervation of retinorecipient nuclei of the thalamus and pretectal area exhibits variability across 24 h

**Figure 1:**
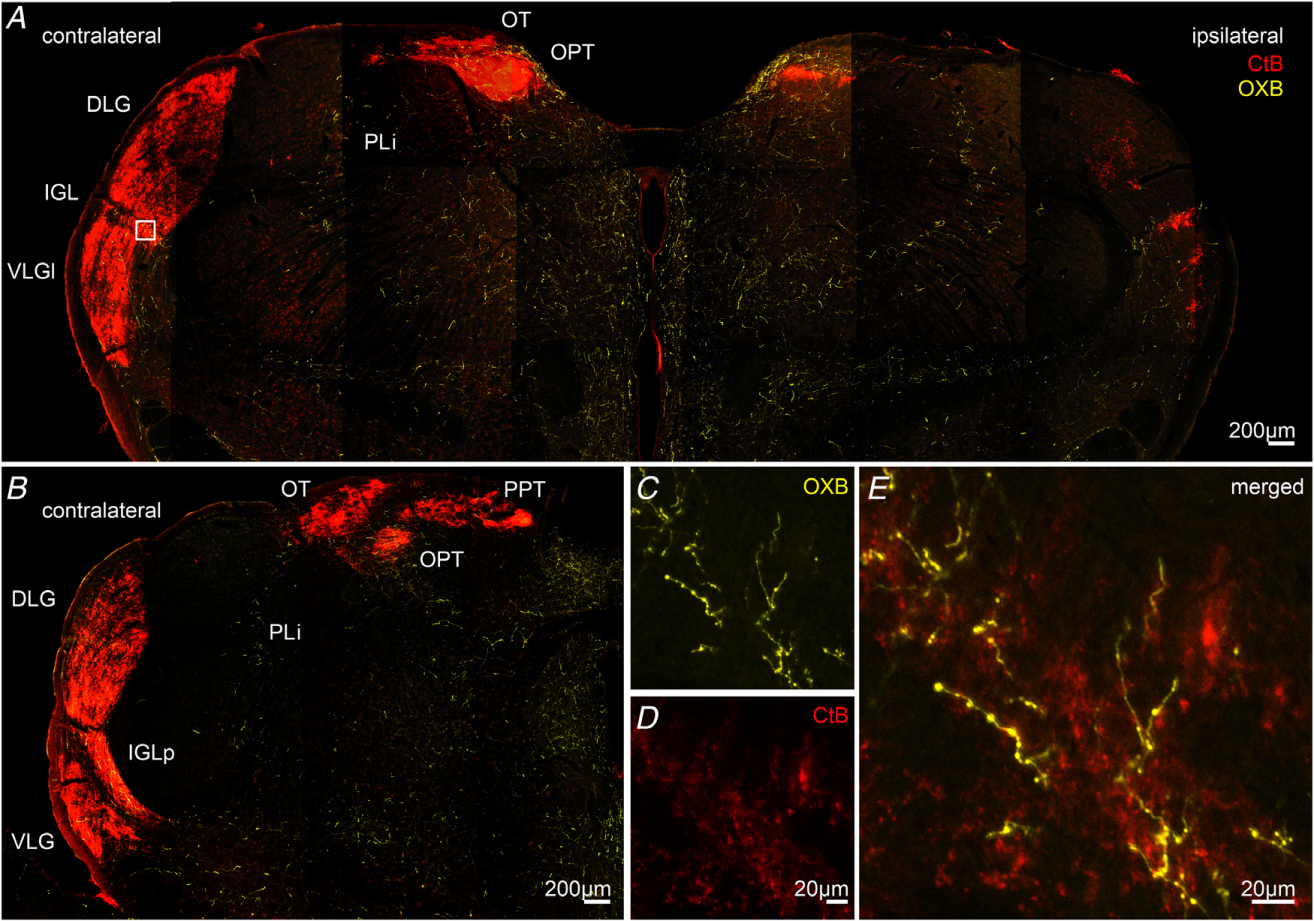
Orexinergic innervation of retinorecipient brain structures in the thalamus and pretectal area *A*, confocal micrograph showing retinal terminals in red (after the intraocular injection of CtB) and OXB‐ir fibres in yellow. Note the dense retinal innervation of the brain nuclei contralateral to the injection site, with some signals found in the ipsilateral OPT and IGL, as well as both the VLG and DLG. The VLG receives retinal innervation in its lateral division only (VLGl). The PLi and nucleus of the optic tract (OT) are innervated almost completely by the contralateral eye. *B*, the same immunohistochemical staining for the posterior part of the LGN and the caudal pretectal complex with the posterior pretectal nucleus (PPT). Note that, in both (*A*) and (*B*), OXB‐ir fibres densely or moderately innervate the IGL, VLG, PLi and OPT (especially its shell) and are sparse in the DLG, OT and PPT. *C*, high magnification of the OXB‐ir fibres in the IGL (the area depicted by a white box in *B*). *D*, high magnification of retinal fibres in the same area. *E*, merged OXB and CtB immunoreactivity. Note the evident difference in fibre morphology. [Color figure can be viewed at wileyonlinelibrary.com]

Previous studies carried out in our laboratory and by others have suggested a functional link between orexinergic and visual systems in rodents (Bayer *et al*. 2004; Webb *et al*. 2008; Liu *et al*. 2011; Pekala *et al*. 2011; Chrobok *et al*. 2016, 2017). To further examine the orexinergic innervation pattern of subcortical visual structures in the rat thalamus and pretectum, we performed intraocular injections of seven rats with CtB and, after 3 days, transcardially perfused them at the beginning of the light (ZT1, *n* = 3) or dark phase (ZT13, *n* = 4). By co‐immunostaining against CtB and OXB, we were able to visualize retinal terminals and orexinergic fibres, respectively (Fig. 1). Further inspection of thalamic/pretectal slices revealed an interesting pattern: OXB‐ir fibres were dense/moderate at the area of retinorecipient structures involved in the non‐visual processing, such as the IGL, ventrolateral geniculate nucleus (VLG), OPT and PLi, and sparse in the DLG or other pretectal areas. No evident co‐localization of CtB immnoreactivity and OXB immnoreactivity was found, with major differences in fibre morphology between these arising from the retina (extremely dense and arborized) and those of the putatively hypothalamic origin (sparser and thick, with varicosities) (Fig. 1*C–E*). No qualitative differences emerged between day and night.

**Figure 2:**
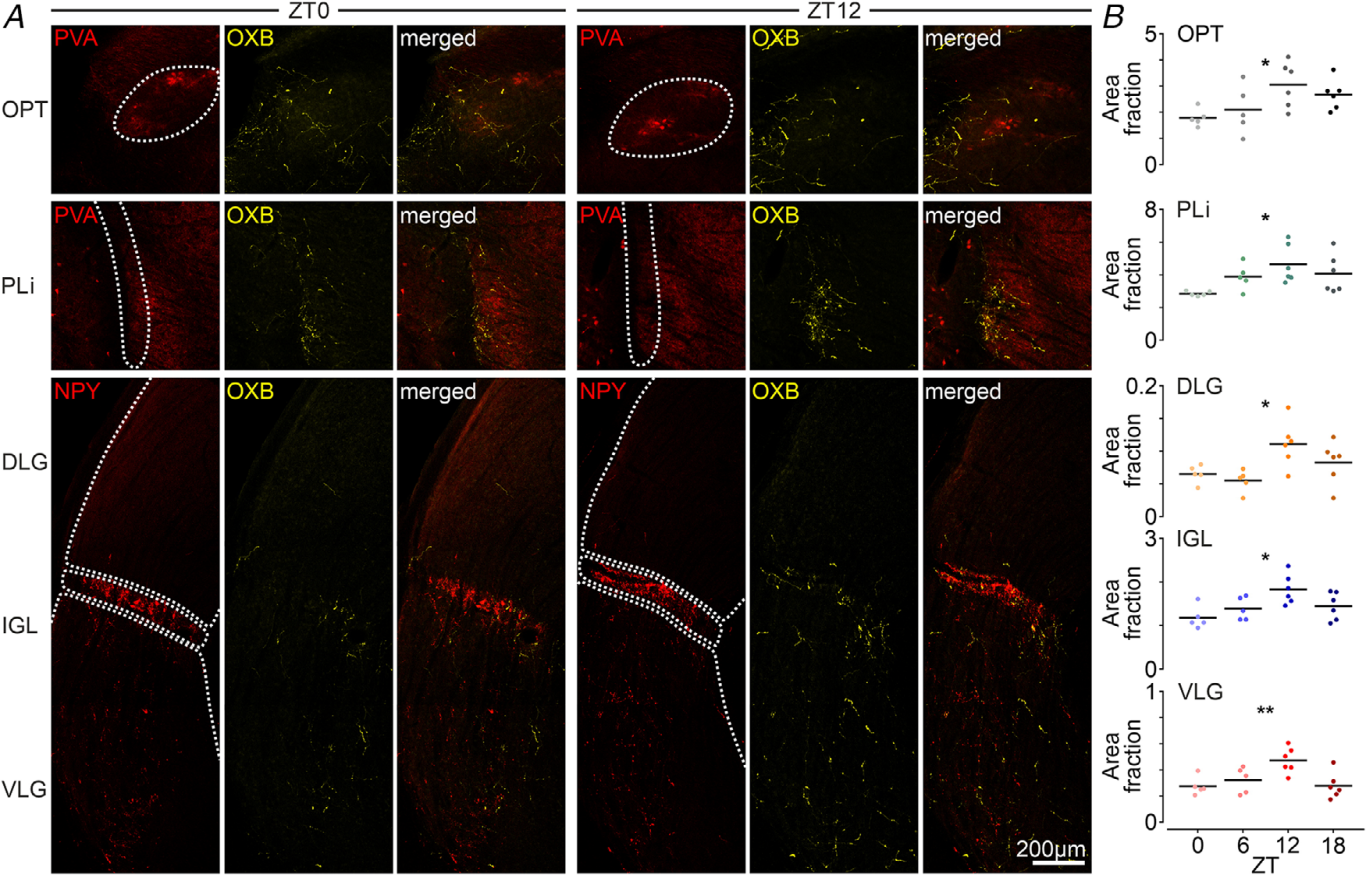
Orexinergic innervation of the subcortical visual structures in the thalamus and pretectal area exhibits day to night variability *A*, confocal micrographs of OXB‐ir fibres (in yellow) at the area of subcortical visual structures, for which borders were outlined by use of marker immunostaining (in red): PVA for the olivary pretectal (OPT) and posterior limitans nuclei (PLi), or NPY, which delineates borders of the IGL dividing the lateral geniculate complex into its DLG and VLG. Images were chosen as representative for the beginning of the light (ZT0, left) and dark (ZT12, right) phases. *B*, significant day to night variability with a nocturnal rise of OXB‐ir fibre density was found in all nuclei tested (OPT: ^*^ *P* = 0.0382; PLi: ^*^ *P* = 0.0433; DLG: ^*^ *P* = 0.0145; IGL: ^*^ *P* = 0.0145; VLG: ^**^ *P* = 0.0058, *n* = 24, one‐way ANOVA). Black bars indicate the mean. [Color figure can be viewed at wileyonlinelibrary.com]

Because the orexinergic system of the hypothalamus exhibits circadian rhythmicity and is sensitive to light conditions (Taheri *et al*. 2000; Deboer *et al*. 2004; Zhang *et al*. 2004; Marston *et al*. 2008; Azeez *et al*. 2018), we subsequently studied OXB‐ir fibres innervating thalamic/pretectal retinorecipient brain areas by transcardial perfusion of 22 rats at four time points (ZT0 *n* = 5, ZT6 *n* = 5, ZT12 *n* = 6 and ZT18 *n* = 6). Evident daily variability of OXB immnoreactivity was found in the OPT, PLi, DLG, IGL and VLG, with the acrophase at the beginning of dark phase and the nadir at light onset (Fig. 2*A*). For a better visualization of studied brain areas, slices were co‐stained for PVA or NPY to mark the boundaries of the OPT or the PLi and IGL, respectively. The densest orexinergic innervation amongst these was observed in the PLi, with the OXB‐ir area fraction increasing from 2.85 ± 0.1 at ZT0 to 4.65 ± 1.2 at ZT12 (*P* = 0.0433, one‐way ANOVA) (Fig. 2*B*). A similar density of OXB‐ir fibres was seen in the OPT, exhibiting a day to night rise from 1.78 ± 0.3 to 3.05 ± 0.9 (*P* = 0.0382, one‐way ANOVA) (Fig. 2*B*). Vast variability among subnuclei was noted in the LGN, where orexinergic axons robustly innervate the IGL, appear in the VLG in moderate proportions and are very sparse in the DLG. However, their OXB immnoreactivity significantly elevates from ZT0 to ZT12 (IGL: 1.18 ± 0.3 to 1.83  ±  0.3, *P* = 0.0145; VLG: 0.28 ± 0.1 to 0.47 ± 0.1, *P* = 0.0058; DLG: 0.065 ± 0.01 to 0.111 ± 0.03, *P* = 0.0145, one‐way ANOVA) (Fig. 2*B*), with overall higher nocturnal levels compared to the day.

**Figure 3:**
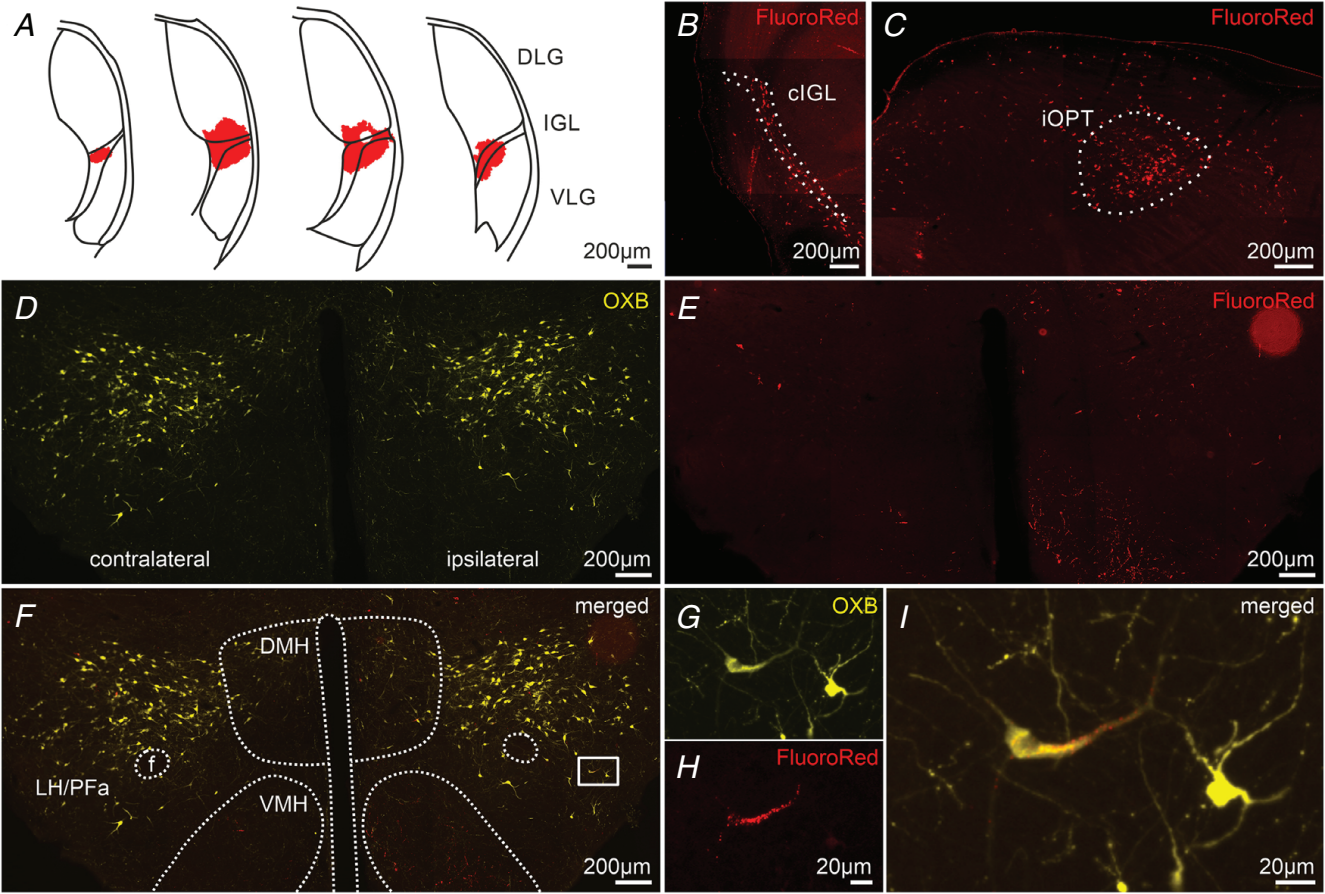
Orexinergic innervation of the LGN arises from the LH/PFa *A*, a reconstruction of the FluoroRed retrograde dye injection into the LGN, centred at the IGL, which distinguishes itself by showing the highest density of orexinergic innervation in the complex. *B*, epifluorescence photomicrograph with numerous cells filled with FluoroRed (in red) in the contralateral IGL (cIGL) to the injection side. *C*, high numbers of FluoroRed‐filled cells in the ipsilateral pretectal area to the injection site, with a robust signal in the posterior division of the ipsilateral olivary pretectal nucleus (iOPT). *D*, example immunohistochemical staining against OXB (in yellow) after colchicine treatment, showing orexinergic cell somata in the LH/PFa and dorsolateral nucleus of the hypothalamus (DMH). *E*, FluoroRed‐positive cells in the hypothalamus. Note single cells in the LH/PFa and DMH and numerous in the ventrolateral nucleus of the hypothalamus (VMH). Most of the FluoroRed‐filled cells were found in the hypothalamus ipsilateral to the injection site. *F*, merged image with the hypothalamic areas outlined. f, fornix. *G*, high magnification of the area delineated by a white rectangle in (*F*), immunostained for OXB. *H*, high magnification of the same area with the FluoroRed fluorescence. *I*, merged image showing the co‐localization of the retrograde dye and OXB immunoreactivity, confirming this orexinergic cell to innervate the ipsilateral LGN. [Color figure can be viewed at wileyonlinelibrary.com]

The results of our CtB intraocular injection experiment suggest that, despite the proposed expression of orexins by retinal ganglion cells, the retino‐thalamic/pretectal transport of these peptides appears to be improbable. Therefore, in the next step, we aimed to establish the hypothalamic source of orexins for retinorecipient brain areas because the lateral hypothalamic/perifornical area (LH and PFa, respectively) is the only known brain locus for orexin synthesis. We performed intra‐LGN injections (centred at the IGL) of FluoroRed (20 nL, right hemisphere) (Fig. 3*A*) and FluoroGreen (20 nL, left hemisphere) retrograde tracers to five adult rats, followed by i.c.v. injections of colchicine (0.1 mg in 5 µL of saline) after 7 days. These procedures allowed us to inspect the granulae of tracer retrogradely transported from the LGN to LH/PFa in orexinergic neurons that accumulated orexins in their cell bodies across the day and night (Fig. 3*D*). Three FluoroGreen and three FluoroRed injections were classified as being located in the LGN. Therefore, all five brains were inspected for co‐localization of the dye and OXB immunoreactivity at the level of cell somata. However rare (up to three per section), we found OXB‐ir cells filled with retrograde tracer scattered across the LH/PF in all brains tested, ipsilateral to the tracer injection site (Fig. 3*D–I*). High numbers of tracer‐positive cells were found in other areas known to innervate the IGL, such as in the contralateral IGL to the injection site (Fig. 3*B*) or in the ipsilateral OPT (Fig. 3*C*). At the level of hypothalamus, high numbers of cells innervating the LGN were noted in the ventromedial nucleus (Fig. 3*E* and *F*).

### Orexin A activates LGN neurons throughout the day and night

Our previous patch clamp and single‐channel extracellular studies *ex vivo* performed on young rats during the light phase showed that all three regions of the LGN are electrophysiologically responsive to orexins (Palus *et al*. 2015; Chrobok *et al*. 2016, 2017). Here, we used MEA recordings *ex vivo* to simultaneously evaluate, on a mass scale, the firing rate, pattern and responsiveness to drugs of single units in all three parts of the LGN.

**Figure 4:**
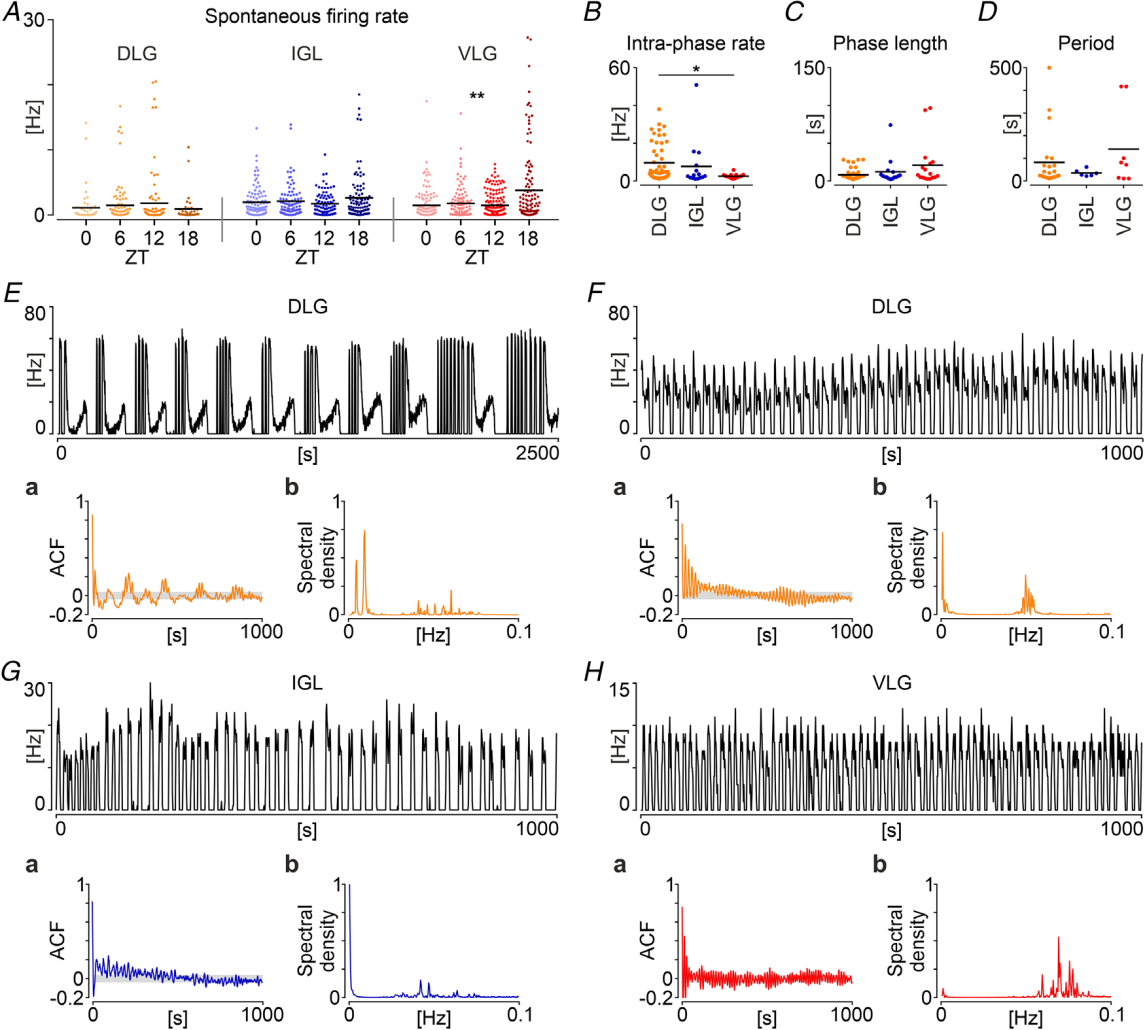
Daily and ultradian firing patterns in the LGN *ex vivo* *A*, variability of firing rate over four time points across 24 h in three parts of the LGN: the DLG (*n* = 261; in shades of yellow), IGL (*n* = 377; blue) and VLG (*n* = 515; red). A significant nocturnal rise was seen in the VLG (*n* = 515, ^**^ *P* = 0.0025, Kruskal–Wallis test). *B*, the frequency of action potential generation measured during the high firing phase for phasic cells in the LGN, which showed significant variability amongst its subareas (*n* = 82, *P* = 0.0185, Kruskal–Wallis test), with a significantly higher rate in the DLG compared to the VLG (^*^ *P* = 0.0193, Dunn's multiple comparisons test). *C*, average length of the high firing phase among the LGN subregions. *D*, the period of rhythmic LGN units in the DLG, IGL and VLG. Black bars indicate the mean. *E*, frequency histogram showing an example recording of the rhythmic unit in the DLG, exhibiting a compound infra‐slow oscillation formed by a train of shorter bursts followed by a longer firing activity period. *F*–*H*, examples of the slow phasic firing in the DLG, IGL and VLG, respectively. *Ea*, *Fa*, *Ga*, *Ha*, autocorrelograms (ACF, autocorrelation factor). *Eb*, *Fb*, *Gb*, *Hb*, periodograms for displayed autocorrelogram examples. Grey shading denotes the 95% confidence interval. [Color figure can be viewed at wileyonlinelibrary.com]

In total, we spike‐sorted 1600 single units located in the LGN, recorded from 20 thalamic slices from 20 rats culled across the light/dark cycle (ZT0, 6, 12 and 18; *n* = 5 per time point). In total, 1153 cells were spontaneously active during the baseline recording, whereas 447 emerged in the response to further application of drugs. Spontaneous firing in the LGN was highly diversified across its subnuclei, with significant day to night variability in firing rates of single units in the VLG (*P* = 0.0025, *n* = 515), but not IGL (*P* = 0.5208, *n* = 377), nor DLG (*P* = 0.3344, *n* = 261, all tested with a Kruskal–Wallis test) (Fig. 4*A*).

Interestingly, around 7% (*n* = 82) of spontaneously active single units across the LGN exhibited ultradian patterns of neuronal firing (Fig. 4*E–H*), a feature most prominent for the DLG (19%, *n* = 50). Overall, phasic units recorded in the DLG distinguished themselves by demonstrating the significantly highest intra‐phase firing rates (*P* = 0.0185, Kruskal–Wallis test) (Fig. 4*B*), although the average length of the phase did not differ amongst the LGN subareas (*P* = 0.366, Kruskal–Wallis test) (Fig. 4*C*). Visual inspection of autocorrelograms enabled us to classify 36 units as rhythmic in the slow/infra‐slow frequency band (periods from periodograms: DLG 82.05 ± 123 s, *n* = 22; IGL 34.83 ± 15 s, *n* = 6; VLG 140.00 ± 174 s, *n* = 6; *P* = 0.8586, Kruskal–Wallis test) (Fig. 4*D*). Again, a majority of rhythmic units was located in the DLG, with six units exhibiting unique and compound firing patterns of a train of shorter bursts preceded by a longer tonic discharge of action potentials (Fig. 4*E*). Spontaneous activity of the remaining units could be classified as irregular or tonic.

Recent evidence suggests that electrophysiological responsiveness of the DLG to orexins falls dramatically in the course of development (Orlowska‐Feuer *et al*. 2019). Hence, we investigated whether the sensitivity to OXA endures in all three parts of the LGN in adult rats across 24 h. Therefore, after 1 h of baseline recording, we applied OXA (200 nm) and recorded neuronal responses in 19 brain slices (one slice at ZT0 was excluded from analysis as a result of technical problems during first drug application). OXA‐evoked excitations were the densest in the IGL, where we recorded 369 excited units out of 219 recording locations (response factor = 1.71). Similarly, a high response density was seen in the VLG, with 633 single‐unit excitations at 419 electrodes (response factor = 1.51). By contrast, responses to OXA were very rare in the DLG, where we recorded 33 activated units at 395 locations (response factor = 0.08).

**Figure 5:**
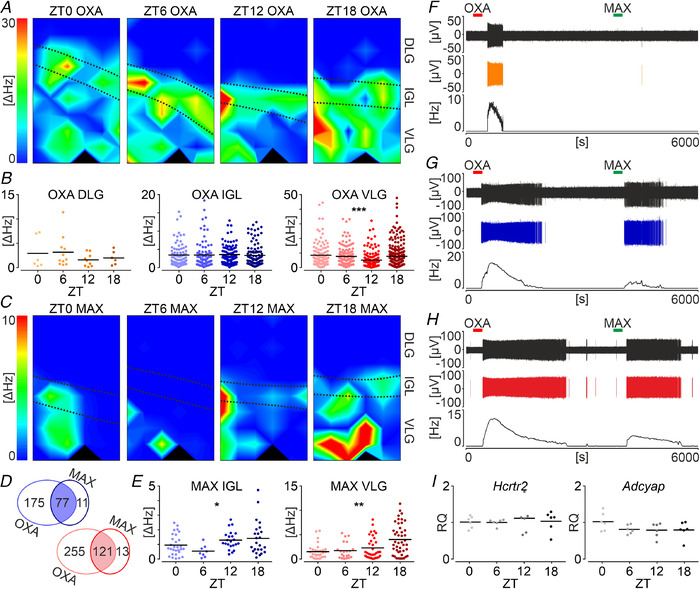
Daily effects of OXA and the PAC1 receptor activation in the LGN *A*, example heatmaps showing the multi‐unit activity change (ΔHz) after the OXA (200 nm) application during a multi‐channel recording of the LGN at four different time points across 24 h. Warm colours code the high excitation. *B*, amplitudes of OXA‐evoked excitation for the DLG (*n* = 33; in shades of yellow), IGL (*n* = 369; blue) and VLG (*n* = 633; red) across 24 h. Day to night variability in the sensitivity to OXA was present in the VLG (*n* = 633, ^***^ *P* = 0.0001, Kruskal–Wallis test). *C*, example heatmaps revealing multi‐unit activity change after maxadilan (MAX, 100 nm; PAC1 receptor agonist) application during a multi‐channel recording of the LGN at four time points across day and night. *D*, the great majority of single units responsive to MAX were also excited by OXA in both the IGL (in blue) and VLG (in red). *E*, daily variability in the amplitude of responses to MAX in the IGL (*n* = 87, ^*^ *P* = 0.0209, Kruskal–Wallis test) and VLG (*n* = 135, ^**^ *P* = 0.0014, Kruskal–Wallis test). *F*–*H*, example recordings from the DLG, IGL and VLG, respectively, showing a dual responsiveness to OXA and MAX. Top to bottom: raw extracellular recording trace, one spike‐sorted single unit and the firing rate histogram (bin = 30 s). Red bars show the treatment with OXA, whereas green bars show treatment with MAX. *I*, stable gene expression of *Adcyap* (coding PAC1 receptor) and *Hcrtr2* (gene for orexin receptor 2) in the whole LGN. RQ, relative gene expression (with reference to ZT0 mean). Black bars indicate the mean. [Color figure can be viewed at wileyonlinelibrary.com]

To address possible day to night differences in the LGN responsiveness to OXA across 24 h, which would co‐occur with a variable presence of the ligand in the orexinergic fibres, we subsequently compared the amplitude of OXA‐evoked responses in each subnucleus amongst four time points (Fig. 5*A*). No significant differences were seen in the DLG, nor IGL (*n* = 33, *P* = 0.6606 and *n* = 369, *P* = 0.3574, respectively, Kruskal–Wallis test) (Fig. 5*B*); these structures proved to exhibit stable responses across 24 h. Evident rhythm was found in the VLG, with the acrophase at ZT0 (8.53 ± 8.5 Hz) and nadir at ZT12 (5.20 ± 5.3 Hz, *n* = 633, *P* = 0.0001, Kruskal–Wallis test) (Fig. 5*B*), which stayed in an anti‐phasic relationship with respect to the day to night variability in OXB‐ir fibre density.

To actively regulate the reception of a circadian input, brain structures may diversify their receptor expression or otherwise shape their responsiveness by various postsynaptic mechanisms (Belle & Piggins, 2017; Chrobok *et al*. 2020). To check the possibility of a daily variation in the orexin receptors expression in the LGN complex, we culled 24 rats at four time points across 24 h and dissected whole bilateral LGNs for molecular studies. Stable expression of *Hcrtr2* (gene for the orexin receptor 2) was observed throughout the day and night (*n* = 24, *P* = 0.8854, one‐way ANOVA) (Fig. 5*I*). Expression of *Hcrtr1*, coding the orexin receptor 1, was almost undetectable (ΔCT values >35).

### Neurons in the IGL and VLG which respond to PAC1 activation are sensitive to OXA

Retinal ganglion cells extensively innervating retinorecipient brain sites involved in the non‐image forming visual functions (such as the IGL, OPT and SCN) express ‘circadian photopigment’ melanopsin and most of them utilize PACAP together with glutamate to excite targeted neurons (Hannibal *et al*. 2000; Hattar *et al*. 2006). To further address our observation regarding the co‐existence of a dense orexinergic and ipRGCs‐derived innervation of studied brain areas, we administered a selective PAC1 receptor agonist maxadilan (MAX, 100 nm) to twelve LGN slices recorded on the MEA, as a second drug application 1 h after OXA treatment (*n* = 3 slices per time point) (Fig. 5*C*). As predicted, no changes in firing rates evoked by MAX were found in the DLG (Fig. 5*F*), which was previously shown not to express PAC1 (Joo *et al*. 2004). By contrast, single units located in the IGL (Fig. 5*G*) and VLG (Fig. 5*H*) exhibited excitatory responses to the drug (IGL: *n* = 87/138 and VLG: *n* = 135/244 recording locations). Interestingly, a marked majority of units that proved sensitive to PAC1 receptor activation were also responsive to OXA in both the IGL (88%) (Fig. 5*D* and *G*) and VLG (90%) (Fig. 5*D* and *H*), suggesting the modulatory action of orexins at most of the ipRGC‐innervated LGN neurons. On the other hand, only 30% of all OXA‐sensitive units in the IGL and 32% in the VLG (Fig. 5*D*) were co‐excited by MAX. This implies that, although OXA acts at almost all neurons responsive to PAC1 receptor activation, the predominant action of the orexinergic system is to excite putatively non‐ipRGC innervated cellular subpopulations of the LGN.

Moreover, the comparison of MAX‐evoked excitation amplitudes at different time points revealed variable sensitivity to PAC1 activation across 24 h. In the IGL, MAX application induced the highest response at ZT18 (1.42 ± 1.2 Hz) and was the least efficient at the opposite time point (ZT6: 0.54 ± 0.4 Hz, *n* = 87, *P* = 0.0209, Kruskal–Wallis test) (Fig. 5*E*). Units in the VLG followed a similar daily pattern, with low responsiveness during the light phase (minimum at ZT0: 1.52 ± 1.5 Hz) and high during the night (maximum at ZT18: 4.02 ± 3.1 Hz, *n* = 135, *P* = 0.0014, Kruskal–Wallis test) (Fig. 5*E*).

Similar to orexin receptors, we examined PAC1 receptor coding gene (*Adcyap*) expression throughout 24 h, which showed a steady level during the day and night (*n* = 24, *P* = 0.1869, one‐way ANOVA) (Fig. 5*I*).

### Orexins act through OX_2_R in the DLG and VLG, whereas they act via both OX_1_R and OX_2_R in the IGL

**Figure 6:**
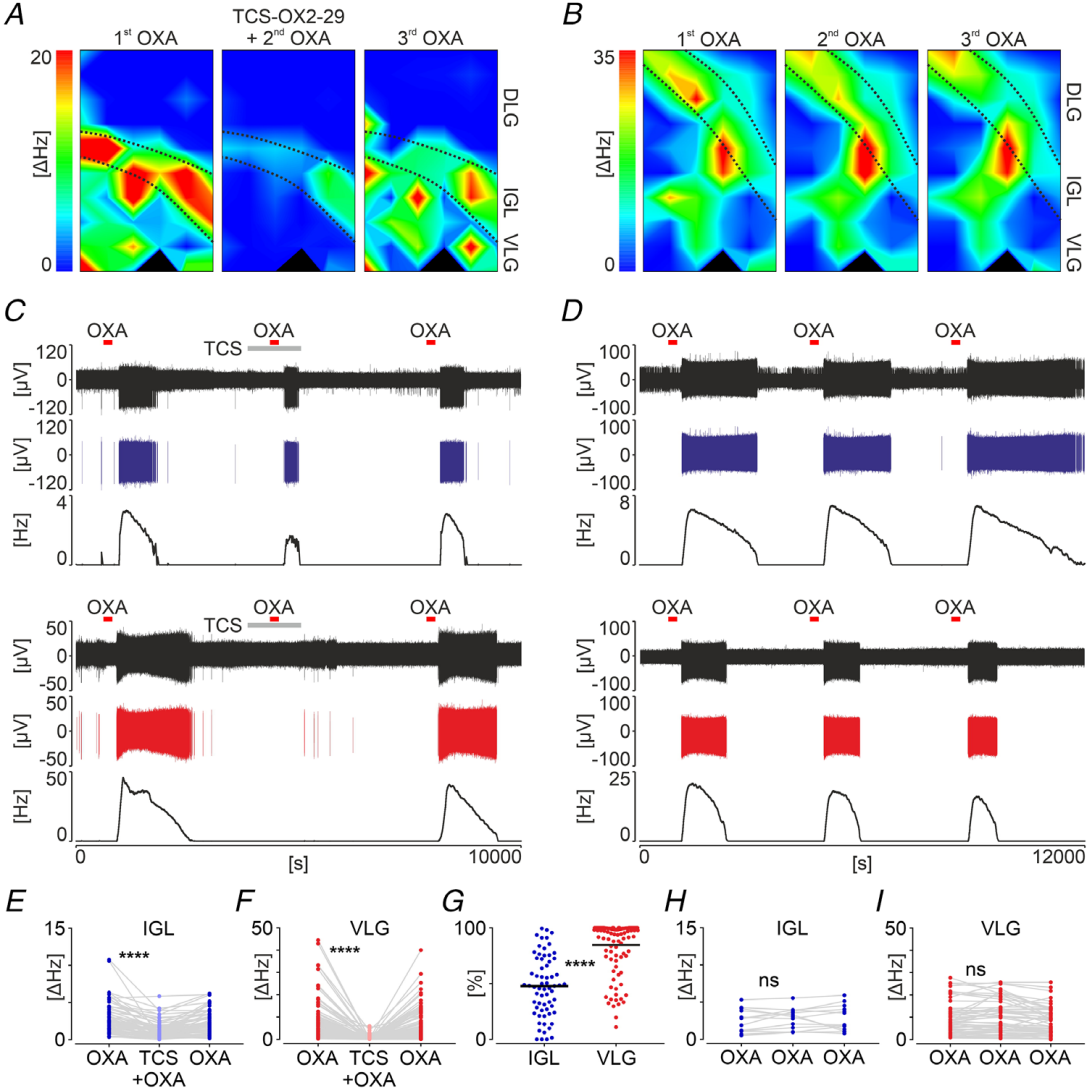
Orexin receptors expression varies across the LGN *A* and *B*, example heatmaps depicting multi‐unit activity change (ΔHz) after OXA (200 nm) application in the LGN recorded on multi‐electrode arrays. *A*, treatment with TCS‐OX2‐29 (10 µm, orexin receptor 2 antagonist). Note that warm colours coding excitations were sustained in the IGL only, when OXA was administered in the presence of the antagonist. *B*, repetitive control OXA applications. *C*, example recording traces from the IGL (in blue) and ventrolateral geniculate nucleus (VLG; in red), illustrating the potency of TCS‐OX2‐29 to antagonize OXA‐evoked excitations. Top to bottom: raw extracellular recording trace, one spike‐sorted single unit and the firing rate histogram (bin = 30 s). The time of TCS‐OX2‐29 treatment is coded by a grey bar and OXA applications are indicated by red bars. *D*, parallel examples of control recordings. *E* and *F*, the change in the amplitude of OXA‐evoked excitation after TCS‐OX2‐29 treatment in the IGL (*n* = 65, *P* < 0.0001, Dunn's test) and VLG (*n* = 106, *P* < 0.0001, Dunn's test), respectively. *G*, a significant difference of the functional orexin receptor type between the IGL and VLG (*n* = 171, *P* < 0.0001, Mann–Whitney test). *H* and *I*, no evident reduction of the OXA‐evoked excitation was seen between control repetitive applications, neither in the IGL (*n* = 13, *P* = 0.6536, Dunn's tests), nor the VLG (*n* = 54, *P* = 0.1212, Dunn's tests), respectively. Black bars indicate the mean. [Color figure can be viewed at wileyonlinelibrary.com]

Orexins exert their electrophysiological actions via two metabotropic G‐protein coupled receptors: OX_1_R and OX_2_R, with different affinity to OXA and OXB (OXA is a dual agonist for both OX_1_R and OX_2_R, whereas OXB has a higher affinity towards OX_2_R), as well as distinct expression sites in the brain, therefore contributing to discrete pathologies in case of their malfunctions (Kukkonen, 2013; Li & de Lecea, 2020). The type of orexin receptor present in the LGN subnuclei has been investigated previously, although with divergent conclusions. Therefore, in the present study, we aimed to resolve this issue by simultaneously recording electrical responses to OXA (200 nm) in all three subparts of the LGN in the presence of a selective OX_2_R antagonist TCS‐OX2‐29 (10 µM) (Fig. 6*A*). Aiming not to favour any specific time of day, this set of experiments was conducted on five LGN slices throughout the day/night cycle (*n* = 2 for ZT6 and *n* = 1 each for ZT0, ZT12 and ZT18) and the results obtained were pooled. In the IGL, treatment with the TCS‐OX2‐29 significantly dampened neuronal responses to OXA (2.79 ± 2.2 Hz *vs*. 1.33 ± 1.2 Hz, *n* = 65, *P* < 0.0001, Dunn's test) (Fig. 6*C* and *E*), but did not completely diminish them. Contrasting results were obtained from recording locations in the VLG, where OX_2_R antagonism almost completely blocked the response to OXA (7.88 ± 8.6 Hz *vs*. 0.59 ± 1.0 Hz, *n* = 106, *P* < 0.0001, Dunn's test) (Fig. 6*C* and *F*). Overall, 47.9 ± 27% of the OXA‐evoked response was abolished in the IGL, whereas 84.7 ± 23% was abolished in the VLG (*n* = 171, *P* < 0.0001, Mann–Whitney test) (Fig. 6*G*). Because responses to OXA are very sparse in the DLG, in this set of recordings, we found only three OXA‐evoked excitations, all of which were effectively antagonized by the TCS‐OX2‐29 treatment (3.71 ± 2.9 Hz *vs*. 0.13 ± 0.15 Hz, *n* = 3, *P* = 0.0286, Dunn's test; data not shown). To confirm that responsiveness to OXA was specifically damped as a result of receptor antagonism and not the time course, we applied OXA for the third time, 1 h after the TCS‐OX2‐29 washout, and observed the restored excitations. Further control was gained by triple, repetitive applications of OXA (200 nm) upon one slice at ZT18 (Fig. 6*G*), with no significant depletion of a second response to the drug (IGL: *n* = 13, *P* = 0.6536, VLG: *n* = 54, *P* = 0.1212, Dunn's tests) (Fig. 6*H* and *I*).

### Retinorecipient neurons in the IGL and VLG are a target of the orexinergic system

**Figure 7:**
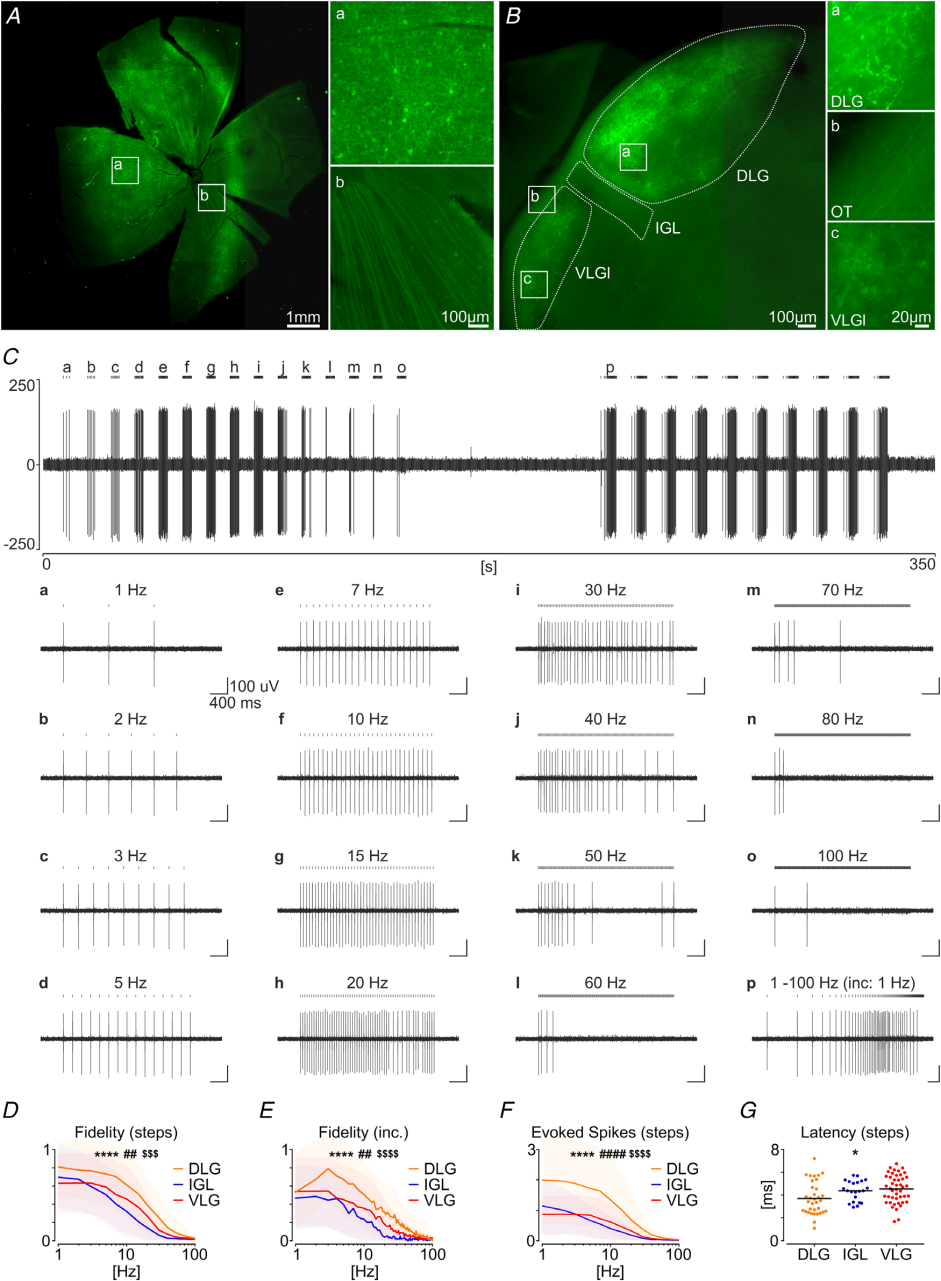
Effects of the optogenetic stimulation of retinal fibres in the LGN *A*, example epifluorescence photomicrograph of a retinal preparation after the intraocular injection of AAV‐Syn‐Chronos‐GFP, with a high magnification of (*Aa*) retinal ganglion cells somata/dendritic arborizations and (*Ab*) axons forming the optic nerve. *B*, thalamic slice containing the LGN, with GFP‐positive axonal projections of the virus‐expressing retinal ganglion cells. High magnifications illustrate axonal terminals at the area of (*Ba*) the DLG, (*Bb*) retinal fibres along the optic tract (OT) and (*Bc*) the retinal innervation of the VLGl. *C*, a raw recording trace showing a single unit responsive to the optogenetical stimulation of retinal fibres in the VLGl. *Ca* to *Co*, responses to 15 different blue light stimulations with a frequency increasing in steps, from 1 to 100 Hz. *Cp*, one trace of the responses to the optogenetic stimulation, with 100 light flashes of an incremental frequency from 1 to 100 Hz. Black vertical bars indicate light flashes (1 ms). *D*, differences in the fidelity tested with the ‘step stimulation’ amongst the LGN subareas (*n* = 102, frequency: ^****^ *P* < 0.0001, structure: ##*P* = 0.0058, interaction: $$$*P* < 0.0002, RM two‐way ANOVA). *E*, differences in the fidelity tested with the ‘incremental stimulation’ protocol (*n* = 102, frequency: ^****^ *P* < 0.0001, structure: ##*P* = 0.0036, interaction: $$$$*P* < 0.0001, RM two‐way ANOVA). *F*, differences in the number of spikes evoked in 10 ms windows tested with the ‘step stimulation’ (*n* = 102, frequency: ^****^ *P* < 0.0001, structure: ###*P* = 0.0001, interaction: $$$$*P* < 0.0001, RM two‐way ANOVA). Shading illustrates the SD. *G*, differences in the latency to spike generation after the blue light flash (*n* = 102, ^*^ *P* = 0.0125, one‐way ANOVA). Colours: yellow, blue and red code the DLG, IGL and VLG, respectively. Black bars indicate the mean. [Color figure can be viewed at wileyonlinelibrary.com]

To directly investigate whether retinorecipient neurons of the LGN receive information from the orexinergic system, we next injected AAV‐Syn‐Chronos‐GFP into a vitreous chamber of one eye in five rats. Subsequently (after 4–5 weeks), we recorded neuronal activity form the contralateral LGN on the MEA. Expression of the viral genes was additionally inspected under an epifluorescence microscope at the level of the retina (injection site) and retinal fibres in the LGN (Fig. 7*A* and *B*). Indeed, optogenetic stimulation of retinal fibres in the LGN evoked profound excitations throughout the complex, at the area clearly correlated with the spread of a virus in the retina. Here, we used two stimulation protocols to determine the retinorecipient abilities of LGN neurons. First, we stimulated slices with brief 1 ms light flashes in 3 s trains of an increasing frequency (‘step stimulation’, from 1 Hz to 100 Hz) (Fig. 7*Ca–Co*). Next, we used ten trains of incremental frequency 1 ms flashes in the same frequency range (‘incremental stimulation’) (Fig. 7*Cp*).

We were able to evoke optogenetic responses in 102 neurons recorded from seven brain slices, where 34 single units were located in the DLG, 22 were located in the IGL and 46 were located in the VLG. Clear differences in the response characteristics were noted amongst these areas. The DLG was characterized by the highest fidelity in both the ‘step’ (*n* = 102, *P* = 0.0058) (Fig 7*D*) and ‘incremental’ stimulation (*n* = 102, *P* = 0.0036) (Fig 7*E*) protocols and the number of evoked spikes was enhanced in the 10 ms time window (*n* = 102, *P* < 0001, all tested with RM two‐way ANOVA) (Fig 7*F*). This LGN subregion also exhibited the shortest latency to spike generation after the blue light flash (*n* = 102, *P* = 0.0125, one‐way ANOVA) (Fig 7*G*).

**Figure 8:**
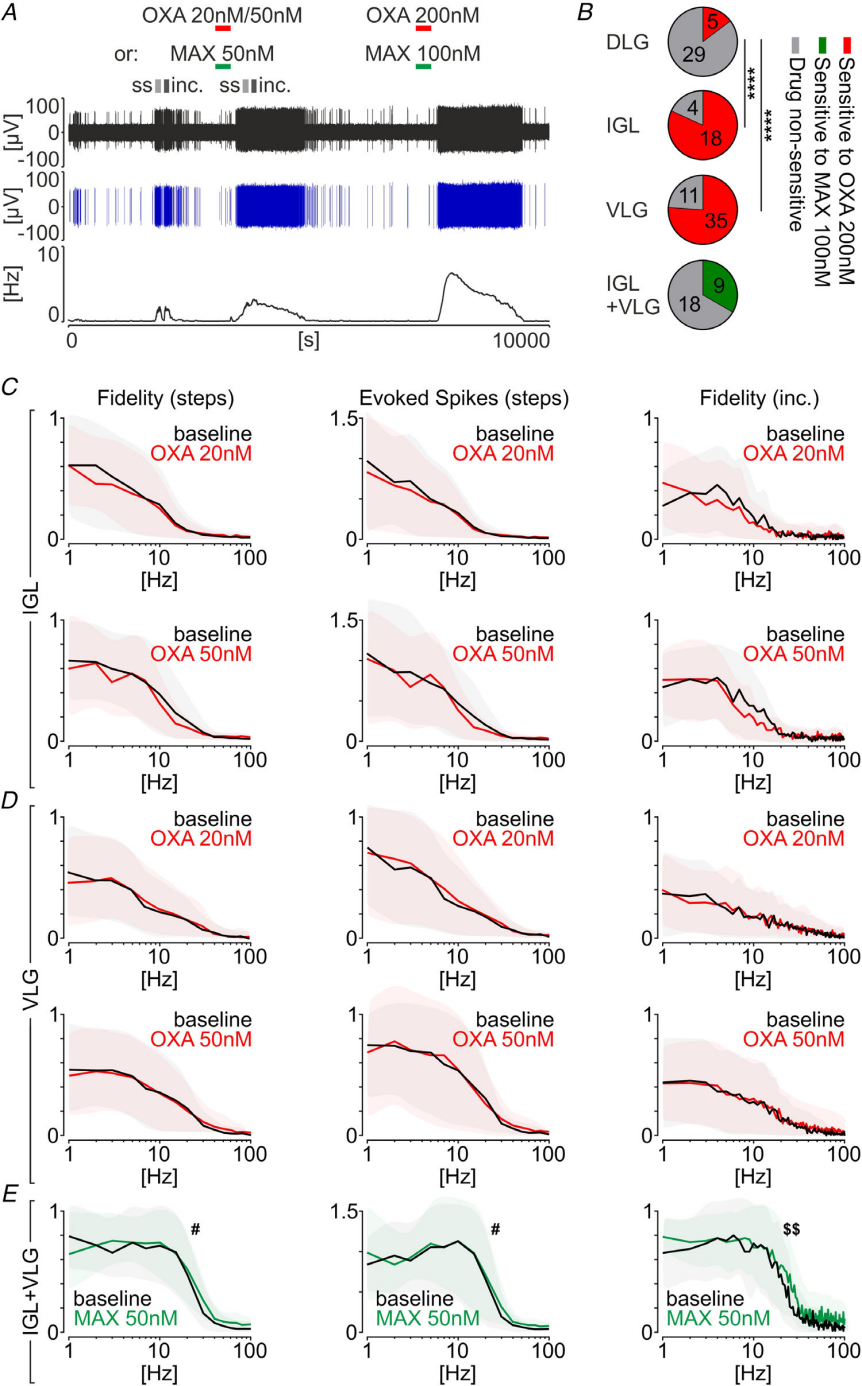
OXA activates retinorecipient neurons in the LGN, but does not directly modulate the optogenetically‐evoked retinal input *A*, an experimental protocol showing a sequence of drug applications and optogenetic stimulations over the example raw extracellular recording trace (in black) showing one spike‐sorted single unit in the ventrolateral geniculate nucleus (VLG, in blue) and a corresponding firing rate histogram (bin: 30 s). Red bars indicate the time of OXA application, whereas green indicates treatment with maxadilan (MAX, a selective PAC1 receptor agonist). Flashes of blue light were performed in two protocols (ss, step stimulations; inc., incremental frequency stimulations). Top to bottom: raw recording trace, spike‐sorted single unit and firing rate histogram (bin = 30 s). *B*, proportions of retinorecipient units sensitive to the drug treatment (VLG *vs*. DLG ^****^ *P* < 0.0001, IGL *vs*. DLG ^****^ *P* < 0.0001, Fisher's tests). Excited by OXA is indicated in red, excited by MAX is indicated in green and non‐responsive is indicated in grey. *C* and *D*, OXA at concentrations of 20 nm and 50 nm does not evoke any change in measured parameters of the response to optogenetic stimulations, in both the IGL and VLG, respectively. *E*, for the IGL and VLG MAX‐responsive units, the activation of PAC1 receptor significantly changed the response to optogenetic stimulations of retinal terminals, by increasing the fidelity (*n* = 9, frequency: *P* < 0.0001, treatment: #*P* = 0.0487, interaction: *P* = 0.0642, RM two‐way ANOVA) and the number of evoked spikes in 10 ms windows after a light flash (*n* = 9, frequency: *P* < 0.0001, treatment: #*P* = 0.0245, interaction: *P* = 0.8717, RM two‐way ANOVA) measured by a ‘step stimulation’ protocol. Additionally, it changed the shape of the light frequency coding in the ‘incremental frequency stimulation’ protocol (*n* = 9, frequency: *P* < 0.0001, treatment: *P* = 0.0570, interaction: $$*P* = 0.0088, RM two‐way ANOVA). Shading indicates the SD. [Color figure can be viewed at wileyonlinelibrary.com]

Subsequently, we repeated optogenetic stimulations of retinal fibres in the presence of OXA (20 nm and/or 50 nm) and/or MAX (50 nm) (Fig. 8*A*). A lower drug concentration was used to avoid overexcitation, which might have disturbed the optogenetic responses. Therefore, at the end of each protocol, we administered OXA at a higher concentration (200 nm) to evoke a clear excitatory response. When examined in single units, which confirmed their responsiveness only, the treatment with 20 nm OXA did not change any measured parameters of the response to blue light flashes either in the IGL (*n* = 12; fidelity ‘step’: *P* = 0.2665, evoked spikes: *P* = 0.3247, fidelity ‘inc.’: *P* = 0.3192) (Fig. 8*C*) or in the VLG (*n* = 23; fidelity ‘step’: *P* = 0.8159, evoked spikes: *P* = 0.3489, fidelity ‘inc.’: *P* = 0.4131) (Fig. 8*D*). A lack of modulation was also true for 50 nm OXA both in the IGL (*n* = 15; fidelity ‘step’: *P* = 0.2350, evoked spikes: *P* = 0.3932, fidelity ‘inc.’: *P* = 0.6301) (Fig. 8*C*) and VLG (*n* = 27; fidelity ‘step’: *P* = 0.8267, evoked spikes: *P* = 0.6947, fidelity ‘inc.’: *P* = 0.1728) (Fig. 8*D*). However, contrasting results were obtained for the treatment with MAX (50 nm). In the IGL and VLG studied together (a total of nine units responsive to higher, 100 nm concentration of MAX), activation of PAC1 receptor during the retinal fibres stimulation significantly increased the fidelity (‘step stimulation’: *n* = 9, *P* = 0.0487) and enhanced the number of spikes following the light flash (*n* = 9, *P* = 0.0245, all measured with RM two‐way ANOVA) (Fig. 8*E*). Treatment with MAX significantly changed the shape of frequency coding by IGL/VLG units, as measured by the incremental frequency protocol (fidelity ‘inc.’: *n* = 9, *P* = 0.0088; RM two‐way ANOVA interaction) (Fig. 8*E*). As a result of an insignificant number of units responsive to OXA in the DLG in this protocol, we were unable to reliably test effects of its modulatory tone upon light responses.

Despite the lack of direct modulatory action of OXA upon retinal responses, we further evaluated the possibility of the retinal information converging with that carried by the orexinergic system. Therefore, we investigated whether these single units, which proved sensitive to the optogenetic stimulation, were also excited by OXA application (evaluated based on the higher concentration: 200 nm). A striking majority (81.8%, *n* = 18/22) (Fig. 8*B*) of retinorecipient units were responsive to OXA in the IGL, with a similarly high proportion of 76.1% (*n* = 35/46) (Fig. 8*B*) in the VLG, although only 14.7% (*n* = 5/34) (Fig. 8*B*) in the DLG (VLG *vs*. IGL *P* = 0.7580, VLG *vs*. DLG *P* < 0.0001, IGL *vs*. DLG *P* < 0.0001, Fisher's tests) (Fig. 8*B*) were responsive. These results highlight the conspicuous inter‐relationship of the systems under investigation. Nine out of 27 opto‐responsive units (33.3%) (Fig. 8*B*) were sensitive to 100 nm MAX application in the IGL/VLG, with no DLG units being excited by PAC1 receptor activation.

## Discussion

The primary findings of the present study are: (i) the orexinergic system with the source in the lateral hypothalamus densely innervates non‐image forming visual structures, with a higher nocturnal orexin availability than during the behaviourally quiescent day; (ii) orexins robustly excite IGL and VLG neurons, in contrast to relatively sparse responsiveness of the DLG in adult rats; (iii) OX_2_R is expressed throughout all three subareas of the LGN, whereas the co‐expression of OX_1_R was seen in the IGL only; (iv) despite the lowered sensitivity to orexins in the VLG at the beginning of the night, the LGN exhibits a stable expression of OX_2_R throughout 24 h; and (v) orexins excite directly retinorecipient neurons in the IGL and VLG, with high convergence of orexinergic and putatively retinal PACAP/PAC1 receptor systems.

### Orexinergic innervation of the subcortical visual system

The orexinergic system of the lateral hypothalamus extensively innervates many areas throughout the brain and spinal cord (Peyron *et al*. 1998). Orexinergic modulation with respect to the mammalian visual system is largely understudied; however, recently, a few studies have emerged linking the arousal promoting orexins with retinorecipient brain areas. First, reports from the SCN showed the high sensitivity of clock cells to the orexin application, with varying mechanisms of action during the circadian day (mostly presynaptic) and night (a direct postsynaptic inhibition) (Belle *et al*. 2014). Intriguingly, orexinergic fibres are sparse in the SCN, and densely innervate the adjacent areas, using volume transmission to exert their modulatory actions on the master clock (Peyron *et al*. 1998; Belle *et al*. 2014). Orexinergic neurons, in return, are controlled by the master clock, with their activity peaking during the night (disinhibited by the SCN), and are responsive to arousing stimuli such as a dark pulse during the light phase (Yoshida *et al*. 2001; Marston *et al*. 2008; Azeez *et al*. 2018). Second, orexins were found to directly excite the primary visual cortex because they generally provoke the high‐spread cortical activation. In this case, orexins activate cortical neurons located in the layer VI, which projects in a feedback loop to the thalamus, predominantly targeting the DLG (Bayer *et al*. 2004; Wenger Combremont *et al*. 2016). Finally, an exceptionally high density of orexinergic fibres was shown in the IGL, with a moderate density for the VLG, as well as a sparse density (yet exhibiting a nocturnal rise) for the DLG (Peyron *et al*. 1998; Pekala *et al*. 2011; Chrobok *et al*. 2016, 2017).

The results of the present study re‐evaluate previous observations, directly showing an apposition of orexinergic and retinal fibres, as is especially evident for these retinorecipient structures, which function as the non‐image forming visual system, such as the IGL, the lateral division of VLG (VLGl), and the PLi or OPT. Interestingly, as reported previously for the DLG (Chrobok *et al*. 2017), the OXB‐ir fibre density follows a daily patterning at the area of all subcortical visual nuclei tested, with a high nocturnal ligand presence. This observation stands in line with the activation of orexinergic neurons during the dark phase or the diurnal fluctuation of prepro‐orexin mRNA and orexin levels in different brain areas, peaking during the wakefulness and arousal‐related behaviours (Taheri *et al*. 2000; Yoshida *et al*. 2001; Karnani *et al*. 2020). Collectively, these results suggest that hypothalamic orexinergic neurons follow a circadian pattern of activity and orexin synthesis, as is also evident at the distant axonal projections, increasing orexin release onto targeted neurons during the night/active behaviour.

### The source of orexinergic innervation for the subcortical visual system

The LH/PFa constitutes the main source of orexins in the central nervous system, with few orexin‐synthesizing neurons in the adjacent dorsolateral nucleus of the hypothalamus (Peyron *et al*. 1998; Nambu *et al*. 1999). However, both orexins and orexin receptors were reported to be expressed in the retina, including retinal ganglion cells, and they plausibly act as local neurotransmitters (Liu *et al*. 2011; Qiao *et al*. 2017; Zhang *et al*. 2018). Here, we show that OXB is most probably not transported along the optic nerve because OXB immunoreactivity was not seen either in the retinal terminals labelled with the intraocular‐injected CtB or in the optic tract. Also, the morphology of orexinergic fibres (with clear varicosities) differs markedly from those of a retinal origin. Hence, we hypothesize that orexins do not undergo anterograde transport mechanisms and do not leave the somato‐dendritic compartment of retinal ganglion cells, unlike other retinal peptides such as vasopressin or PACAP (Hannibal *et al*. 2000; Tsuji *et al*. 2017).

Indeed, our tract tracing study revealed orexinergic neurons of the LH/PFa to innervate the LGN. As a result of the complex and elongated shape of the LGN, we were unable to efficiently fill the whole nucleus with the tracer without affecting adjacent and subjacent structures (e.g. the medial geniculate nucleus or zona incerta), also innervated by orexinergic neurons (Peyron *et al*. 1998). Therefore, we centred our injections at the IGL, comprising a part of the LGN localized in a middle of a complex, also being a major target of orexinergic fibres. The inability to quantitatively describe orexinergic cells innervating the whole LGN constituted a limitation to this approach; however, in return, we were able to reliably and selectively pinpoint these neurons that target the confined injection area. The experiments reported here propose that the hypothalamus (and not the retina) is a source of orexinergic innervation for the LGN, suggesting the same for the subcortical visual system as whole.

### Daily and ultradian variability of neuronal firing in the LGN

The firing rate and pattern of LGN neurons *in vivo* can be shaped by their intrinsic membrane properties, a major excitatory input from the retina dependent on the ambient light levels and modulatory tones from a plethora of neurotransmitter systems, or, most probably, a balancing combination of these. The electrophysiological experiments performed in the present study were carried out *ex vivo*, where most of the network mechanisms and distant inputs are ablated during the slice preparation. Therefore, we were able to extract the spontaneous activity of neurons populating three distinct parts of the LGN, stemming from their intrinsic membrane properties, limited local network mechanisms and/or long‐lasting effects of *in vivo* challenges before the cull. In our study, we did not observe evident persistent excitatory effects of light upon the single unit activity in the LGN *ex vivo* in animals culled at four time points across the light/dark cycle; in contrast, there was a significant nocturnal increase in the spontaneous firing, constrained to the VLG only. Because the VLG is not implicated in the image forming vision, but rather in visuomotor and circadian functions (Harrington, 1997), this intrinsic rise of spontaneous firing probably does not underlie the previously postulated circadian modulation of vision (Barlow, 2001). Instead, it may either be a reflexion of the night‐time upregulated excitation coming from the arousal‐promoting brainstem nuclei or an intrinsic mechanism helping the bare nocturnal retinal input to regulate non‐image forming functions during the behaviourally active night.

Growing evidence suggests that the action potential generation pattern is alongside the firing rate an important and parallel channel of the information flow in the central nervous system, particularly in the sensory systems where it codes additional stimulus characteristics (Braun *et al*. 1994; Buzsaki, 2004; Buzsáki, 2006). Distinct oscillations of the rat LGN activity were found *in vivo*, where different frequency bands shape an outcome activity pattern. Most common and structured are the retinal‐derived rhythms, with an infra‐slow and gamma band oscillation coding ambient light levels (Lewandowski & Błasiak, 2004; Storchi *et al*. 2017; Chrobok *et al*. 2018). However, both of these are lost *ex vivo*, with most LGN neurons exhibiting tonic firing (Blasiak & Lewandowski, 2004; Chrobok *et al*. 2018). Intrinsic slow oscillations of membrane potential and firing were also reported in the cat and rodent DLG, similar to these reported in the present study (Hughes *et al*. 2002; Zhu *et al*. 2006). Here, as a result of simultaneous recording of three parts of the LGN, we compare the ultradian patterning of firing amongst their neurons, showing that the DLG possesses the most evident oscillatory abilities *ex vivo*. These experiments reveal diversified and complex neurophysiological characteristics of the LGN on different timescales from seconds to hours, with most pronounced ultradian rhythmicity of the DLG and day to night variability in the VLG.

### Electrophysiological modulation of the LGN by orexins

The orexinergic modulation of the LGN electrical activity was previously studied in our laboratory, as well as by others. Primary studies were carried out on the IGL, as a result of its functional interlink with the biological timing system (Webb *et al*. 2008). It has been hypothesized that orexins acting on the NPY‐utilizing neurons, which connect the IGL to SCN, may transmit arousal‐induced phase shifts and circadian resetting (Webb *et al*. 2014). Indeed, *ex vivo* studies have revealed orexins to excite IGL neurons, including these synthesizing NPY (Palus *et al*. 2015). Intriguingly, not only is the geniculo‐hypothalamic pathway activated at the level of IGL, but also OXA was found to potentiate the NPY‐evoked inhibition of SCN clock cells and circadian phase shifting (Belle *et al*. 2014). Recent evidence clearly demonstrates this pathway to transmit arousal and metabolic information from the food‐entrainable oscillator system to shift the phase of a master clock, as required for the proper food anticipatory activity (Saderi *et al*. 2013; Fernandez *et al*. 2020). The results of our detailed study confirm the robust responsiveness of the IGL to OXA, which remains steady throughout 24 h. Thereby, it suggests IGL neurons to be ready to equivocally respond to the information carried by the orexinergic excitation across the day and night, without any active mechanisms shaping its sensitivity in a day to night manner. We can only hypothesize that, *in vivo*, with an indifferent sensitivity to orexins and higher nocturnal orexin release, the IGL neurons are more excited by these peptides during the active phase.

Opposing results were obtained for the VLG, which exhibits daily variability of sensitivity to OXA, staying in an anti‐phasic relationship with respect to that of OXB immunoreactivity in the terminals innervating this structure. This phenomenon may be explained by the partial receptor internalization or desensitization as a result of the high ligand concentration during the dark phase, rather than an evident change in the receptor gene expression, which was not detected in our qPCR measurements performed at the whole LGN tissue. Additionally, the anti‐phasic relationship between ligand presence and responsiveness may provide a compensatory mechanism for maintaining stable excitation throughout the light/dark cycle. Here, we also confirm that sensitivity to OXA persists in adult animals both in the IGL and VLG, which is important because previous experiments on these systems were carried out in young rats (Palus *et al*. 2015; Chrobok *et al*. 2016).

The responsiveness of the DLG to the orexin application was highly controversial in previous studies. First, two independent groups claimed the insensitivity to orexins of the DLG and other ‘specific’ thalamic nuclei, in general (Bayer *et al*. 2002; Govindaiah & Cox, 2006). Subsequently, our patch clamp *ex vivo* study performed on 2–3‐week‐old rat pups on the thalamo‐cortical neurons in the DLG showed widespread excitation evoked by OXA in ∼75% of the cells tested (Chrobok *et al*. 2017). However, these results were not replicated in the single‐unit extracellular study on adult rats *in vivo*, nor in patch clamp *ex vivo* recordings on brain slices obtained from ∼6‐week‐old rats, pinpointing possible developmental changes in the DLG reaction to the modulation carried by the orexinergic system (Orlowska‐Feuer *et al*. 2019). To address this issue over a larger scale, in the present study, we examined responses to OXA at almost 400 recording locations in the DLG of adults rats, across 24 h. Although neuronal responses were evident, they were very rare (found at only ∼8% of all recording sites). The present study adds to the current understanding of an orexin action in the DLG decreasing with age, although the exact mechanisms of this developmental change remain unknown.

### Orexin receptors differentiate three subparts of the LGN

OXA and OXB bind to orexin receptors with different affinity (OX_1_R is ∼50 fold more selective towards OXA than OXB, whereas OX_2_R has a similar affinity to both peptides), and OX_1_R and OX_2_R are expressed in distinct brain areas (Kukkonen, 2013; Thompson *et al*. 2014; Li & de Lecea, 2020). Structures such as the laterodorsal tegmental nucleus, pedunculopontine tegmental nucleus or ventral tegmental area were found to co‐express both orexin receptors, whereas the locus coeruleus preferentially expresses OX_1_R and the tubero‐mammillary nucleus ‐ OX_2_R (Hervieu *et al*. 2001; Marcus *et al*. 2001; Inutsuka & Yamanaka, 2013; Ch'ng & Lawrence, 2015). In the LGN, a specific expression pattern was assigned to each subnucleus. OX_2_R was suggested to be predominantly expressed in the VLG and DLG (Chrobok *et al*. 2016, 2017). In the IGL, initial studies proposed OX_1_R to be dominant (Marcus *et al*. 2001), although a subsequent electrophysiological report described OX_2_R to be prevailing (Pekala *et al*. 2011). Here, in line with our previous patch clamp study (Palus *et al*. 2015), we demonstrate both receptors to be functional in the IGL, in contrast to the DLG and VLG responding to OXA via OX_2_R alone.

### Orexins target retinorecipient neurons of the IGL and VLG

Previous indirect evidence suggested a possibility of the orexinergic system modulating retinorecipient neurons: (i) OXA was found to excite thalamo‐cortical neurons of the DLG, which are majorly retinorecipient (Chrobok *et al*. 2017); (ii) close appositions of OXA‐ir fibres with putatively retinorecipient enkephalinergic neurons of the IGL were shown (Pekala *et al*. 2011); and (iii) OXA was established as exciting the putatively light‐responsive nitric oxide synthase‐positive neurons in the VLGl (Chrobok *et al*. 2016). However, our optogenetic study is the first to provide direct evidence indicating that OXA excites a prominent majority of retinorecipient cells in both IGL and VLG (precisely in the retinorecipient VLGl).

To further address this problem, our study aimed to unravel a possible convergence of two peptidergic systems in the LGN: the orexinergic system of the lateral hypothalamus and the putatively retinal PACAP/PAC1 receptor system. PACAP was shown to be expressed in the retina by ipRGCs co‐expressing melanopsin, as well as to be released at the retinal terminals in the subcortical visual system to act upon PAC1 receptors (Hannibal & Fahrenkrug, 2004; Juhl *et al*. 2007). Therefore, in the present study, we used a selective PAC1 receptor agonist, and not PACAP itself, to exclude its binding to VPAC1 and VPAC2 receptors, also expressed in the LGN (Joo *et al*. 2004). Because PAC1 is not expressed in the DLG (coincident with a very sparse innervation by ipRGCs) (Hannibal & Fahrenkrug, 2004; Joo *et al*. 2004), no changes in the electrical activity after an agonist application were seen in this brain area. Yet, robust excitatory responses were evoked in the IGL and VLG, almost exclusively at neurons also activated by OXA.

Surprisingly, the amplitude of neuronal responses to PAC1 receptor activation was not equal at the four time points tested across 24 h, instead exhibiting a significant daily variance, with peak responsiveness in the middle of the night for both the IGL and VLG. Here, we also show that *Adcyap* (a gene coding PAC1 receptor) has a fixed expression throughout 24 h. Thus, these changes may stem from the otherwise cyclic availability of the ligand (abundantly released during the light phase) possibly causing PAC1 receptors to internalize, or other active non‐receptor mechanisms shaping the overall electrical response. Together with our observation that the PAC1 receptor agonist strengthens the response to optogenetic stimulation of retinal fibres, we suggest its role in the sharpening of the LGN sensitivity to light during the behaviourally active dark phase, when light availability is strongly limited. Overall, for the ipRGCs‐recipient parts of the LGN, the sensibility to orexinergic modulation is more pronounced during the day, in contrast to nocturnally more potent PACAP.

## Conclusions

In conclusion, we report the robust excitatory effects of OXA on the electrical activity of the LGN, with a clear distinction from the less responsive DLG and highly sensitive IGL and VLG. For the first time, the present study directly demonstrates that the orexinergic system of the lateral hypothalamus targets retinorecipient neurons, additionally displaying high convergence with a putatively retinal PACAP/PAC1 receptor system. Moreover, we unravel the day to night variability in the orexinergic innervation of the subcortical visual system, together with daily variation in the responsiveness to OXA in the VLG. Our study provides a new basis for understanding the physiological link between the orexinergic and visual systems and proposes orexins to relay arousal‐related activation for retinorecipient brain centres in a circadian fashion.

## Additional information

### Competing interests

The authors declare that they have no competing interests.

### Author contributions

LC, JSJL and MHL conceived the study. LC supervised the project and provided financial support. LC designed, carried out, analysed and interpreted electrophysiological studies. MW wrote custom R scripts and analysed the baseline MEA data. KP and LC designed optogenetic experiments. KP wrote custom Spike2 and MatLab scripts and optimized the automated spike‐sorting for the MultiChannel Systems data. LC performed intraocular injections with help from JDK. JDK and LC obtained the brain tissue for qPCR and LC performed the RNA isolation. MB designed, performed and analysed qPCR experiments. LC, JSJL and JDK performed and analysed the results of immunohistochemical studies. JSJL carried out tract tracing surgeries with help from LC LC and MK performed the confocal imaging. LC wrote the article with critical input from all coauthors, who agreed to the final version submitted for publication.

### Funding

This work was financially supported by the project grant ‘Sonatina II’ 2018/28/C/NZ4/00099 from the Polish National Science Centre. The research was carried out using the equipment purchased through financial support from the European Regional Development Fund in the framework of the Polish Innovation Economy Operational Program (contract no. POIG.02.01.00‐12‐023/08).

## Supporting information


**Statistical Summary Document**

## Data Availability

The data that support the findings of this study are available from the corresponding author upon reasonable request.
