# Supplementary material for: Circadian actions of orexins on the retinorecipient lateral geniculate complex in rat
Source: J Physiol. 2020 Oct 14;599(1):231–52. doi: 10.1113/JP280275 (PMC7821336; doi:10.1113/JP280275)
Supplement: Supplementary file 1 — Statistical Summary Document [file TJP-599-231-s001.docx]

**Manuscript Title:** Circadian actions of orexins on the retinorecipient lateral geniculate complex in rat

**Authors:** Chrobok Lukasz, Jeczmien-Lazur Jagoda Stanislawa, Pradel Kamil, Klich Jasmin Daniela, Bubka Monika, Wojcik Michal, Kepczynski Mariusz, Lewandowski Marian Henryk

**Animal model used, if applicable:** Rat

**Underlying hypothesis:** This investigation tests the hypothesis that orexins provide circadian information to the subcortical visual system, namely the lateral geniculate nucleus of the thalamus (LGN) and studies the daily changes in their electrophysiological action upon single LGN neurons and orexinergic innervation pattern.

**Definitions of ‘n’:**

Question 1, 2, 8: n = animals

Question 3: n = successful unilateral dye injections / rats

Question 4-7, 9: n = individual neurons / *ex vivo* brain slices (n rats = n slices)

Question 10-11: n = n = individual neurons / *ex vivo* brain slices / rats

**Statistical summary table:**

| Experimental question number* | Finding/ conclusion | Experimental location/ variable | Mean value | SD | n val. | P** | Units | Data comparisons | Statistical test | Any other variable | Figure | Comments |
| --- | --- | --- | --- | --- | --- | --- | --- | --- | --- | --- | --- | --- |
| 1. Co-localisation of retinal and OXB-ir fibres (stained with CtB)? | No co-localisation | ZT1 | - | - | 3 | - | - | OXB-ir and CtB-ir | observation | 10-12 week old | 1 | No qualitative differences between ZT0 and ZT13 - no co-localisation in both groups |
|  |  | ZT13 | - | - | 4 | - | - |  |  |  |  |  |
| 2. Daily variability in OXB-ir fibre density? | Significant variability across 24h | OPT ZT0 | 1.78 | 0.3 | 5 | **0.0382** | fraction | ZT | 1way ANOVA | 12-16 week old | 2 | Each brain area was tested separately |
|  |  | OPT ZT6 | 2.09 | 0.9 | 5 |  |  |  |  |  |  |  |
|  |  | OPT ZT12 | 3.05 | 0.9 | 6 |  |  |  |  |  |  |  |
|  |  | OPT ZT18 | 2.68 | 0.6 | 6 |  |  |  |  |  |  |  |
|  |  | PLi ZT0 | 2.85 | 0.1 | 5 | **0.0433** | fraction | ZT | 1way ANOVA |  |  |  |
|  |  | PLi ZT6 | 3.88 | 0.8 | 5 |  |  |  |  |  |  |  |
|  |  | PLi ZT12 | 4.65 | 1.2 | 6 |  |  |  |  |  |  |  |
|  |  | PLi ZT18 | 4.07 | 1.2 | 6 |  |  |  |  |  |  |  |
|  |  | DLG ZT0 | 0.065 | 0.01 | 5 | **0.0145** | fraction | ZT | 1way ANOVA |  |  |  |
|  |  | DLG ZT6 | 0.054 | 0.02 | 5 |  |  |  |  |  |  |  |
|  |  | DLG ZT12 | 0.111 | 0.03 | 6 |  |  |  |  |  |  |  |
|  |  | DLG ZT18 | 0.083 | 0.03 | 6 |  |  |  |  |  |  |  |
|  |  | IGL ZT0 | 1.18 | 0.3 | 5 | **0.0145** | fraction | ZT | 1way ANOVA |  |  |  |
|  |  | IGL ZT6 | 1.39 | 0.3 | 5 |  |  |  |  |  |  |  |
|  |  | IGL ZT12 | 1.83 | 0.3 | 6 |  |  |  |  |  |  |  |
|  |  | IGL ZT18 | 1.45 | 0.1 | 6 |  |  |  |  |  |  |  |
|  |  | VLG ZT0 | 0.28 | 0.1 | 5 | **0.0058** | fraction | ZT | 1way ANOVA |  |  |  |
|  |  | VLG ZT6 | 0.32 | 0.1 | 5 |  |  |  |  |  |  |  |
|  |  | VLG ZT12 | 0.47 | 0.1 | 6 |  |  |  |  |  |  |  |
|  |  | VLG ZT18 | 0.28 | 0.1 | 6 |  |  |  |  |  |  |  |

| 3. The LGN innervation by OXB-ir cells in the LH? | Co-localisation of retrograde dye and OXB-ir | LH/PFa | - | - | 6/5 | - | - | OXB-ir and retrograde dye | observation | 250-350 g | 3 | Observation of every third slice containing OXB-ir cell bodies |
| --- | --- | --- | --- | --- | --- | --- | --- | --- | --- | --- | --- | --- |
| 4. Daily variability in spontaneous neuronal activity? | Significant variability in the VLG only | DLG ZT0 | 1.12 | 2.7 | 50/5 | 0.3344 | Hz | ZT | Kruskal-Wallis  test | 10 -12 week old | 4A | Each brain area was tested separately |
|  |  | DLG ZT6 | 1.49 | 3.2 | 87/5 |  |  |  |  |  |  |  |
|  |  | DLG ZT12 | 1.82 | 4.5 | 86/5 |  |  |  |  |  |  |  |
|  |  | DLG ZT18 | 0.93 | 2.2 | 37/5 |  |  |  |  |  |  |  |
|  |  | IGL ZT0 | 1.96 | 2.3 | 100/5 | 0.5208 | Hz | ZT | Kruskal-Wallis  test |  |  |  |
|  |  | IGL ZT6 | 2.12 | 2.5 | 89/5 |  |  |  |  |  |  |  |
|  |  | IGL ZT12 | 1.72 | 1.8 | 86/5 |  |  |  |  |  |  |  |
|  |  | IGL ZT18 | 2.64 | 3.6 | 101/5 |  |  |  |  |  |  |  |
|  |  | VLG ZT0 | 1.50 | 2.4 | 116/5 | **0.0025** | Hz | ZT | Kruskal-Wallis  test |  |  |  |
|  |  | VLG ZT6 | 1.79 | 2.4 | 114/5 |  |  |  |  |  |  |  |
|  |  | VLG ZT12 | 1.48 | 1.9 | 159/5 |  |  |  |  |  |  |  |
|  |  | VLG ZT18 | 3.78 | 5.8 | 126/5 |  |  |  |  |  |  |  |
| 5. Variability in phasic pattern among LGN areas? | Significant variability in the intra-phase rate only | DLG | 9.70 | 10.0 | 50/5 | **0.0185** | Hz | Intra-phase rate among areas | Kruskal-Wallis  test | 10 -12 week old | 4B | - |
|  |  | IGL | 7.78 | 12.8 | 15/5 |  |  |  |  |  |  |  |
|  |  | VLG | 2.57 | 1.1 | 17/5 |  |  |  |  |  |  |  |
|  |  | DLG | 8.22 | 7.1 | 50/5 | 0.366 | s | Phase length among areas | Kruskal-Wallis  test |  | 4C | - |
|  |  | IGL | 12.28 | 18.1 | 15/5 |  |  |  |  |  |  |  |
|  |  | VLG | 20.93 | 29.3 | 17/5 |  |  |  |  |  |  |  |
|  |  | DLG | 82.05 | 123.3 | 22/5 | 0.8586 | s | Period among areas | Kruskal-Wallis  test |  | 4D | - |
|  |  | IGL | 34.83 | 14.9 | 6/5 |  |  |  |  |  |  |  |
|  |  | VLG | 140.00 | 174.4 | 8/5 |  |  |  |  |  |  |  |
| 6. Daily variability in the amplitude of response to OXA? | Significant variability in the VLG only | DLG ZT0 | 2.93 | 3.3 | 6/4 | 0.6606 | ΔHz | ZT | Kruskal-Wallis  test | 10 -12 week old | 5B | Each brain area was tested separately |
|  |  | DLG ZT6 | 3.16 | 3.3 | 11/5 |  |  |  |  |  |  |  |
|  |  | DLG ZT12 | 1.60 | 1.3 | 9/5 |  |  |  |  |  |  |  |
|  |  | DLG ZT18 | 2.00 | 1.4 | 6/5 |  |  |  |  |  |  |  |
|  |  | IGL ZT0 | 3.45 | 3.1 | 86/4 | 0.3574 | ΔHz | ZT | Kruskal-Wallis  test |  |  |  |
|  |  | IGL ZT6 | 3.47 | 3.7 | 88/5 |  |  |  |  |  |  |  |
|  |  | IGL ZT12 | 3.60 | 2.7 | 91/5 |  |  |  |  |  |  |  |
|  |  | IGL ZT18 | 3.36 | 3.1 | 102/5 |  |  |  |  |  |  |  |
|  |  | VLG ZT0 | 8.53 | 8.5 | 108/4 | **0.0001** | ΔHz | ZT | Kruskal-Wallis  test |  |  |  |
|  |  | VLG ZT6 | 7.74 | 6.7 | 129/5 |  |  |  |  |  |  |  |
|  |  | VLG ZT12 | 5.20 | 5.3 | 156/5 |  |  |  |  |  |  |  |
|  |  | VLG ZT18 | 7.76 | 8.0 | 240/5 |  |  |  |  |  |  |  |
| 7. Daily variability in the amplitude of response to MAX? | Significant variability in both the IGL and VLG | IGL ZT0 | 0.95 | 0.7 | 29/3 | **0.0209** | ΔHz | ZT | Kruskal-Wallis  test | 10 -12 week old | 5E | Each brain area was tested separately |
|  |  | IGL ZT6 | 0.54 | 0.4 | 8/3 |  |  |  |  |  |  |  |
|  |  | IGL ZT12 | 1.29 | 0.6 | 22/3 |  |  |  |  |  |  |  |
|  |  | IGL ZT18 | 1.42 | 1.2 | 24/3 |  |  |  |  |  |  |  |
|  |  | VLG ZT0 | 1.52 | 1.5 | 31/3 | **0.0014** | ΔHz | ZT | Kruskal-Wallis  test |  |  |  |
|  |  | VLG ZT6 | 1.74 | 1.7 | 21/3 |  |  |  |  |  |  |  |
|  |  | VLG ZT12 | 2.29 | 2.3 | 33/3 |  |  |  |  |  |  |  |
|  |  | VLG ZT18 | 4.02 | 3.1 | 44/3 |  |  |  |  |  |  |  |
| 8. Daily variability in the receptor expression? | No variability | *Hcrtr2* ZT0 | 1.01 | 0.2 | 6 | 0.8854 | RQ | ZT | 1way ANOVA | 12 week old | 5I | Each transcript was tested separately |
|  |  | *Hcrtr2* ZT6 | 1.00 | 0.1 | 6 |  |  |  |  |  |  |  |
|  |  | *Hcrtr2*ZT12 | 1.11 | 0.4 | 6 |  |  |  |  |  |  |  |
|  |  | *Hcrtr2* ZT18 | 1.03 | 0.3 | 6 |  |  |  |  |  |  |  |
|  |  | *Adcyap* ZT0 | 1.03 | 0.3 | 6 | 0.1869 | RQ | ZT | 1way ANOVA |  |  |  |
|  |  | *Adcyap* ZT6 | 0.81 | 0.1 | 6 |  |  |  |  |  |  |  |
|  |  | *Adcyap* ZT12 | 0.79 | 0.2 | 6 |  |  |  |  |  |  |  |
|  |  | *Adcyap* ZT18 | 0.80 | 0.2 | 6 |  |  |  |  |  |  |  |
| 9. OX_2_R involved in the response to OXA? | Yes, in both the IGL and VLG; significantly more in the VLG | IGL OXA control | 2.79 | 2.2 | 65/5 | **<0.0001**  **/**  **<0.0001** | ΔHz | Treatment | Kruskal-Wallis test  /  Dunn's test | 10 -12 week old | 5E | Data from all ZT pulled together |
|  |  | IGL OXA + TCS-OX2-29 | 1.33 | 1.2 | 65/5 |  |  |  |  |  |  |  |
|  |  | IGL OXA washout | 2.23 | 1.6 | 65/5 |  |  |  |  |  |  |  |
|  |  | VLG OXA control | 7.88 | 8.6 | 106/5 | **<0.0001**  **/**  **<0.0001** | ΔHz | Treatment | Kruskal-Wallis test  /  Dunn's test |  | 5F |  |
|  |  | VLG OXA + TCS-OX2-29 | 0.59 | 1.0 | 106/5 |  |  |  |  |  |  |  |
|  |  | VLG OXA washout | 6.13 | 6.7 | 106/5 |  |  |  |  |  |  |  |
|  |  | IGL % blocked by TCS | 47.80 | 27.4 | 65/5 | **<0.0001** | % | Antagonist efficiency | Mann-Whitney test |  | 5G |  |
|  |  | VLG % blocked by TCS | 84.72 | 23.2 | 106/5 |  |  |  |  |  |  |  |
|  |  | IGL OXA control 1 | 2.50 | 1.6 | 13/1 | 0.1462  /0.6536 | ΔHz | Treatment | Kruskal-Wallis test / Dunn's test |  | 5H |  |
|  |  | IGL OXA control 2 | 2.79 | 1.4 | 13/1 |  |  |  |  |  |  |  |
|  |  | IGL OXA control 3 | 2.82 | 1.7 | 13/1 |  |  |  |  |  |  |  |
|  |  | VLG OXA control 1 | 9.34 | 7.3 | 54/1 | **0.0003**  /0.1212 | ΔHz | Treatment | Kruskal-Wallis test / Dunn's test |  | 5I |  |
|  |  | VLG OXA control 2 | 10.13 | 7.3 | 54/1 |  |  |  |  |  |  |  |
|  |  | VLG OXA control 3 | 8.19 | 6.2 | 54/1 |  |  |  |  |  |  |  |
| 10. Differences in the response to optogenetic stimulation of retinal terminals among LGN areas? | Yes | DLG | 0.39 | 0.3 | 34/7/5 | **0.0002 /<0.0001 /0.0058** | - | Fidelity (steps) | RM 2way ANOVA: interaction /frequency /area | 10 week old | 7D | Cull at ZT1 |
|  |  | IGL | 0.25 | 0.3 | 22/7/5 |  |  |  |  |  |  |  |
|  |  | VLG | 0.30 | 0.3 | 46/7/5 |  |  |  |  |  |  |  |
|  |  | DLG | 0.18 | 0.2 | 34/7/5 | **<0.0001 /<0.0001 /0.0036** | - | Fidelity  (inc.) | RM 2way ANOVA: interaction /frequency /area |  | 7E |  |
|  |  | IGL | 0.07 | 0.1 | 22/7/5 |  |  |  |  |  |  |  |
|  |  | VLG | 0.11 | 0.1 | 46/7/5 |  |  |  |  |  |  |  |
|  |  | DLG | 0.87 | 0.8 | 34/7/5 | **<0.0001 /<0.0001 /<0.0001** | no. | Evoked spikes (steps) | RM 2way ANOVA: interaction /frequency /area |  | 7F |  |
|  |  | IGL | 0.35 | 0.4 | 22/7/5 |  |  |  |  |  |  |  |
|  |  | VLG | 0.39 | 0.4 | 46/7/5 |  |  |  |  |  |  |  |
|  |  | DLG | 3.70 | 1.4 | 34/7/5 | **0.0125** | s | Latency (steps) | 1way ANOVA |  | 7G |  |
|  |  | IGL | 4.38 | 0.9 | 22/7/5 |  |  |  |  |  |  |  |
|  |  | VLG | 4.54 | 1.3 | 46/7/5 |  |  |  |  |  |  |  |
| 11. Changes in optogenetic response after OXA or MAX? | No, after OXA | IGL control | 0.21 | 0.2 | 12/8/4 | **<0.0001** /0.2665 /0.2794 | - | Fidelity (steps) | RM2way ANOVA: frequency /area /interaction | 10 week old | 8C | Cull at ZT1 |
|  |  | IGL OXA 20nM | 0.19 | 0.2 | 12/8/4 |  |  |  |  |  |  |  |
|  |  | IGL control | 0.27 | 0.3 | 12/8/4 | **<0.0001** /0.3247 /0.9077 | no. | Evoked spikes (steps) | RM2way ANOVA: frequency /area /interaction |  |  |  |
|  |  | IGL OXA 20nM | 0.25 | 0.3 | 12/8/4 |  |  |  |  |  |  |  |
|  |  | IGL control | 0.062 | 0.09 | 12/8/4 | **<0.0001** /0.3192 /**<0.0001** | - | Fidelity (inc.) | RM2way ANOVA: frequency /area /interaction |  |  |  |
|  |  | IGL OXA 20nM | 0.059 | 0.08 | 12/8/4 |  |  |  |  |  |  |  |
|  |  | IGL control | 0.26 | 0.3 | 15/6/3 | **<0.0001** /0.2350 /0.4655 | - | Fidelity (steps) | RM2way ANOVA: frequency /area /interaction |  |  |  |
|  |  | IGL OXA 50nM | 0.24 | 0.3 | 15/6/3 |  |  |  |  |  |  |  |
|  |  | IGL control | 0.36 | 0.4 | 15/6/3 | **<0.0001** /0.3932 /0.4454 | no. | Evoked spikes (steps) | RM2way ANOVA: frequency /area /interaction |  |  |  |
|  |  | IGL OXA 50nM | 0.33 | 0.4 | 15/6/3 |  |  |  |  |  |  |  |
|  |  | IGL control | 0.082 | 0.12 | 15/6/3 | **<0.0001** /0.6301 /**<0.0001** | - | Fidelity (inc.) | RM2way ANOVA: frequency /area /interaction |  |  |  |
|  |  | IGL OXA 50nM | 0.078 | 0.11 | 15/6/3 |  |  |  |  |  |  |  |
|  |  | VLG control | 0.21 | 0.2 | 23/8/4 | **<0.0001** /0.8159 /0.4916 | - | Fidelity (steps) | RM2way ANOVA: frequency /area /interaction |  | 8D |  |
|  |  | VLG OXA 20nM | 0.22 | 0.2 | 23/8/4 |  |  |  |  |  |  |  |
|  |  | VLG control | 0.25 | 0.2 | 23/8/4 | **<0.0001** /0.3489 /0.9141 | no. | Evoked spikes (steps) | RM2way ANOVA: frequency /area /interaction |  |  |  |
|  |  | VLG OXA 20nM | 0.27 | 0.3 | 23/8/4 |  |  |  |  |  |  |  |
|  |  | VLG control | 0.080 | 0.08 | 23/8/4 | **<0.0001** /0.4131 /0.1007 | - | Fidelity (inc.) | RM2way ANOVA: frequency /area /interaction |  |  |  |
|  |  | VLG OXA 20nM | 0.084 | 0.08 | 23/8/4 |  |  |  |  |  |  |  |
|  |  | VLG control | 0.25 | 0.2 | 27/6/3 | **<0.0001** /0.8267 /0.8667 | - | Fidelity (steps) | RM2way ANOVA: frequency /area /interaction |  |  |  |
|  |  | VLG OXA 50nM | 0.25 | 0.2 | 27/6/3 |  |  |  |  |  |  |  |
|  |  | VLG control | 0.34 | 0.3 | 27/6/3 | **<0.0001** /0.6947 /0.9727 | no. | Evoked spikes (steps) | RM2way ANOVA: frequency /area /interaction |  |  |  |
|  |  | VLG OXA 50nM | 0.35 | 0.3 | 27/6/3 |  |  |  |  |  |  |  |
|  |  | VLG control | 0.093 | 0.11 | 27/6/3 | **<0.0001** /0.1728 /**0.0065** | - | Fidelity (inc.) | RM2way ANOVA: frequency /area /interaction |  |  |  |
|  |  | VLG OXA 50nM | 0.112 | 0.10 | 27/6/3 |  |  |  |  |  |  |  |
|  | Yes, after MAX | IGL+VLG control | 0.41 | 0.3 | 9/2/2 | **<0.0001** /**0.0487** /0.0642 | - | Fidelity (steps) | RM2way ANOVA: frequency /area /interaction |  | 8E |  |
|  |  | IGL+VLG MAX 50nM | 0.44 | 0.3 | 9/2/2 |  |  |  |  |  |  |  |
|  |  | IGL+VLG control | 0.55 | 0.5 | 9/2/2 | **<0.0001** /**0.0245** /0.8717 | no. | Evoked spikes (steps) | RM2way ANOVA: frequency /area /interaction |  |  |  |
|  |  | IGL+VLG MAX 50nM | 0.59 | 0.4 | 9/2/2 |  |  |  |  |  |  |  |
|  |  | IGL+VLG control | 0.20 | 0.2 | 9/2/2 | **<0.0001** /0.0570 /**0.0088** | - | Fidelity (inc.) | RM2way ANOVA: frequency /area /interaction |  |  |  |
|  |  | IGL+VLG MAX 50nM | 0.26 | 0.2 | 9/2/2 |  |  |  |  |  |  |  |
